# Reduced C9orf72 expression exacerbates polyGR toxicity in patient iPSC-derived motor neurons and a Type I protein arginine methyltransferase inhibitor reduces that toxicity

**DOI:** 10.3389/fncel.2023.1134090

**Published:** 2023-04-17

**Authors:** Therese L. Dane, Anna L. Gill, Fernando G. Vieira, Kyle R. Denton

**Affiliations:** ALS Therapy Development Institute, Watertown, MA, United States

**Keywords:** amyotrophic lateral sclerosis (ALS), frontotemporal dementia (FTD), C9orf72, protein arginine methyltransferase (PRMT), asymmetric dimethylation, dipeptide repeat protein (DPR), poly-Glycine-Arginine (polyGR), hexanucleotide repeat expansion (HRE)

## Abstract

**Introduction:**

Intronic repeat expansions in the *C9orf72* gene are the most frequent known single genetic causes of amyotrophic lateral sclerosis (ALS) and frontotemporal dementia (FTD). These repeat expansions are believed to result in both loss-of-function and toxic gain-of-function. Gain-of-function results in the production of toxic arginine-rich dipeptide repeat proteins (DPRs), namely polyGR and polyPR. Small-molecule inhibition of Type I protein arginine methyltransferases (PRMTs) has been shown to protect against toxicity resulting from polyGR and polyPR challenge in NSC-34 cells and primary mouse-derived spinal neurons, but the effect in human motor neurons (MNs) has not yet been explored.

**Methods:**

To study this, we generated a panel of C9orf72 homozygous and hemizygous knockout iPSCs to examine the contribution of C9orf72 loss-of-function toward disease pathogenesis. We differentiated these iPSCs into spinal motor neurons (sMNs).

**Results:**

We found that reduced levels of C9orf72 exacerbate polyGR15 toxicity in a dose-dependent manner. Type I PRMT inhibition was able to partially rescue polyGR15 toxicity in both wild-type and C9orf72-expanded sMNs.

**Discussion:**

This study explores the interplay of loss-of-function and gain-of-function toxicity in C9orf72 ALS. It also implicates type I PRMT inhibitors as a possible modulator of polyGR toxicity.

## 1. Introduction

The most frequent known causes of amyotrophic lateral sclerosis (ALS) and frontotemporal dementia (FTD) are mutations in the *C9orf72* gene, which account for 40% of familial and 5% of sporadic ALS cases, and act as a main genetic cause of FTD (DeJesus-Hernandez et al., 2011; Renton et al., 2011; Majounie et al., 2012; Suh et al., 2015; Van Mossevelde et al., 2017). These mutations manifest as expansions of a repeating hexanucleotide (GGGGCC) sequence in the first intron of the *C9orf72* gene (DeJesus-Hernandez et al., 2011; Renton et al., 2011). Translation of the *C9orf72* hexanucleotide repeat expansion mutation (C9-HRE) is facilitated by an unconventional process termed repeat-associated non-AUG (RAN) translation (Ash et al., 2013; Mori et al., 2013). The role of the C9-HRE in ALS and FTD pathogenesis has been explored in the context of three possible disease mechanisms: loss-of-function, or decreased expression of endogenous C9orf72 protein, toxic gain-of-function of mutant sense and antisense repeat RNA, and toxic gain-of-function of RAN-translated dipeptide repeat proteins (DPRs): poly-Glycine-Arginine (polyGR), poly-Proline-Arginine (polyPR), poly-Glycine-Alanine (polyGA), poly-Glycine-Proline (polyGP), and poly-Proline-Alanine (polyPA) (Yang et al., 2020). A body of evidence surrounding each of these mechanisms introduces the possibility of a multiple-hit model, whereby loss-of-function and gain-of-function consequences might simultaneously contribute to C9-HRE-linked neurodegeneration (Braems et al., 2020).

Decreased expression of endogenous C9orf72 protein has been documented in both patient tissue samples and patient-derived induced pluripotent stem cell (iPSC) lines harboring the C9-HRE (Waite et al., 2014; Sivadasan et al., 2016; Frick et al., 2018). Resulting loss in the reported function of the C9orf72 protein has been associated with losses in axonal trafficking (Farg et al., 2014; Gendron and Petrucelli, 2018; Shi et al., 2018). In one study, heterozygous and homozygous deletion of C9orf72, as well as antisense oligonucleotide (ASO)-mediated knockdown, decreased survival of human iPSC-derived MNs, which was ameliorated with exogenous application of C9orf72 protein (Shi et al., 2018). Other studies using *in vitro C9orf72* knockdown and knockout models have also demonstrated dysfunctional autophagic (Sellier et al., 2016; Webster et al., 2016; Yang et al., 2016; Aoki et al., 2017; Fomin et al., 2018; Leskelä et al., 2019), lysosomal (Amick et al., 2016; Aoki et al., 2017; Shi et al., 2018), and endosomal (Farg et al., 2014) behaviors. These autophagy deficits have also been observed in murine models deficient in C9orf72 (O’Rourke et al., 2016; Sullivan et al., 2016). Despite this, much evidence has been collected to assert that loss of endogenous C9orf72 protein alone is not sufficient to induce the neurodegeneration characteristic of ALS and FTD, instead, a combination of loss-of-function and gain-of-function is more likely (Braems et al., 2020; Pal et al., 2021).

Significant cytotoxic gain-of-function effects resulting from exposure to the arginine-rich DPRs (R-DPRs) polyGR and polyPR have also been extensively documented (Freibaum and Taylor, 2017). In *in vitro* systems, indications of detrimental cellular effects by R-DPRs have been detected in HEK293T cells, NSC-34 cells, iPSC-derived cortical and motor neurons, and others (Kwon et al., 2014; Wen et al., 2014; Tao et al., 2015; Lee et al., 2016; Kramer et al., 2018). We have previously reported that NSC-34 cells differentiated to exhibit more neuron-like characteristics are more sensitive to polyGR and polyPR challenge, potentially implicating neuronal cell physiology in R-DPR mechanisms of toxicity (Gill et al., 2019). Further, we have observed the abrogation of R-DPR cytotoxic effects in NSC-34 cells when exogenous R-DPR challenge is administered alongside a co-dose of small-molecule asymmetric (Type I) Protein Arginine Methyltransferase (PRMT) inhibitors (Premasiri et al., 2020; Gill et al., 2021). One study has approached an intersection between DPR toxicity and endogenous C9orf72 loss-of-function, where using an siRNA knockdown model of C9orf72 in GT1-7 and HEK293 cell models, the investigators reported that reduced C9orf72 expression substantially increased neuronal cell death induced by polyGR, polyGA, and polyGP accumulation, when the DPRs were expressed under their natural start codons (Boivin et al., 2020). Taken together, these studies implicate R-DPR toxicity in gain-of-function deficits, and even begin to associate changes in relationship to loss-of-function models. Our previous study links these DPR gain-of-function toxicity outcomes to PRMT activity, suggesting protein arginine methylation state of involved proteins may relate to how resulting toxicity manifests. However, none of the studies mentioned explore the intersection between all three: R-DPR gain-of-function, C9orf72 loss-of-function, and protein arginine methylation regulation.

Some findings point to a possible link between protein arginine methylation and C9orf72 protein activity in ALS models. One study has detailed the role of symmetric (Type II) arginine dimethylation in the association between C9orf72 and ALS-linked proteins p62, SMN, and FUS, which together initiate a cascade to recognize stress granules for autophagic degradation (Chitiprolu et al., 2018). It is also feasible that endogenous C9orf72 protein itself might be modified by PRMTs. The reported function of human C9orf72 is a guanine nucleotide exchange factor (GEF), and protein arginine methylation of another key human GEF, Vav1, has been reported to regulate its localization and function (Blanchet et al., 2005; Lawson et al., 2007; Peng and Wong, 2017). Other findings associate protein arginine methylation with R-DPR gain-of-function toxicity. One study has correlated the accumulation of symmetrically dimethylated polyGR in C9orf72 ALS patient post-mortem brain tissue with longer disease duration and age at death (Gittings et al., 2020). Further, protein arginine methylation activity has recently been implicated in ALS biomarker development. For example, higher levels of asymmetric dimethylarginine in cerebral spinal fluid (CSF) and serum have been shown to correlate faster progression of ALS symptoms (Ikenaka et al., 2019; Ikenaka et al., 2022). Consistent with these results, asymmetrically (Type I) dimethylated forms of polyGR detected in C9orf72 patient brain tissue did not correlate with later disease onset or longer disease duration, in contrast to symmetrically (Type II) dimethylated forms of polyGR, which did (Gittings et al., 2020). These studies associate Type I PRMT activity with worsening ALS disease outcomes, and even with C9orf72 R-DPR gain-of-function phenotypes. However, none of these studies have addressed how endogenous C9orf72 loss-of-function, R-DPR gain-of-function, and protein arginine methylation might all simultaneously contribute to negative outcomes in C9orf72-ALS models.

To explore this, we generated a panel of isogenic *C9orf72* knockout iPSC lines using a CRISPR/Cas9 dual-guide approach, including one hemizygous patient-derived iPSC line with one copy of C9orf72 knocked out, and two homozygous iPSC lines with both copies of C9orf72 knocked out. Genotyping analysis, quantitative reverse transcription polymerase chain reactions (qRT-PCRs), and western blotting methods were used to confirm successful gene editing. These lines were differentiated into sMNs, and exogenous polyGR_15_ was titrated onto cells. Both hemizygous and homozygous *C9orf72* knockout lines, as well as a patient line with the HRE, were the more sensitive to the polyGR_15_ challenge compared to wild-type. After confirming iPSC-derived sMNs with fewer copies of *C9orf72* were more sensitive to polyGR_15_ challenge, we then introduced Type I PRMT inhibition to this assay framework to see how it might affect cytotoxic endpoints. We titrated the Type I PRMT inhibitor MS023 onto VACHT-tdTomato iPSC-derived sMNs concurrently with polyGR_15_ challenge, and measured resulting metabolic capacity at 24 and 48 h using a CellTiter-Blue assay. Results indicated that the Type I PRMT inhibitor MS023 significantly increased cell viability in response to polyGR_15_ challenge at doses from 0.3 to 3 μM as compared to cells treated with polyGR_15_ alone at both endpoints. This dose range required for cell rescue is consistent with previous results using the same dosing scheme in NSC-34 neuron-like mouse cells (Premasiri et al., 2020). These results begin to assess the intersections between PRMT activity, gain-of-function of arginine-rich C9-DPRs, and C9orf72 loss-of-function phenotypes in *C9orf72* ALS models. Future study will explore the possible interplay between these three mechanisms, and their contributions to C9-linked neurodegeneration, further.

## 2. Materials and methods

### 2.1. iPSC cell culture

Cells were cultured in NutriStem hPSC XF Medium (ReproCell USA, Inc., Beltsville, MD, USA, 01–0005) on growth factor reduced Matrigel hESC-qualified matrix (Corning Incorporated – Life Sciences, Tewksbury, MA, USA, 354230). Cells were passaged every 4–7 days by rinsing once with Dulbecco’s phosphate-buffered Saline (DPBS) without calcium and magnesium (Thermo Fisher Scientific, Waltham, MA, USA, 14–190–250) then treated with pre-warmed Accutase (Stemcell Technologies, Cambridge, MA, USA, 07920) for 3 min in 37°C and 5% CO_2_ for dissociation. Dissociated cells were collected and centrifuged at 200 × g for 5 min. PBS was aspirated and the cell pellet was resuspended in NutriStem prior to replating.

### 2.2. Motor neuron differentiation and cell culture

Spinal motor neurons were generated from iPSCs following a previously reported protocol (Du et al., 2015). In short, iPSCs were plated on Matrigel-coated plates in NutriStem with 10 μM rho-associated protein kinase (ROCK) inhibitor (Tocris, Bio-Techne Corporation, Minneapolis, MN, USA, 1254) on day −1. The following day, the media was replaced with base neural differentiation media [1:1 Neurobasal:DMEM-F12 (Gibco USA, Jenks, OK, USA, 11330032), 1X NEAA (Gibco USA, Jenks, OK, USA, 11140050), 1X PenStrep (Gibco USA, Jenks, OK, USA, 15140163), 1X Glutamax (Gibco USA, Jenks, OK, USA, 35050079), 100 μM ascorbic acid (Sigma-Aldrich, Burlington, MA, USA, A4403-100MG), 0.5X N2 (Life Technologies, Carlsbad, CA, USA, A1370701), 0.5X B27 (Life Technologies, Carlsbad, CA, USA, 17504044) supplemented with 2 μM SB431542 (Selleck Chemicals, Houston, TX, USA, S1067), 2 μM DMH1 (Selleck Chemicals, Houston, TX, USA, S7146), 3 μM CHIR-99021 (Axon MedChem, Reston, VA, USA, 1386)]. Media was replaced every other day until day 6, when cells were passaged with Dispase-II (EMD Millipore, Burlington, MA, USA, SCM133) and plated at a 1–6 ratio. The media above was supplemented with 0.1 μM retinoic acid (Reprocell USA, Inc., Beltsville, MD, USA, 04–0021) and 0.5 μM purmorphamine (Reprocell USA, Inc., Beltsville, MD, USA, 04–0009). On day 12 of differentiation, cells were passaged again with Dispase-II and motor neuron progenitor spheres were formed on ultra-low attachment dishes (Corning Incorporated – Life Sciences, Tewksbury, MA, USA, 4615) in base NDM supplemented with 0.5 μM retinoic acid and 0.1 μM purmorphamine. On day 19, MNP spheres were dissociated with Accutase and plated on laminin/poly-ornithine coated plates in base NDM supplemented with 0.5 μM retinoic acid, 0.1 μM purmorphamine, 10 ng/mL CNTF (Peprotech US, Cranbury, NJ, USA, 450–13–100 μg), 10 ng/mL BDNF (R&D Systems, Inc., Minneapolis, MN, USA, 248-BDB-250/CF), 10 ng/mL GDNF (R&D Systems, Inc., Minneapolis, MN, USA, 212-GD-050), and 1 μM 5-Fluoro-2’-deoxyuridine (Sigma-Aldrich, Burlington, MA, USA, F0503). Media was replaced every 2–3 days using an EL406 automated plate washer (Agilent Technologies, Inc., Santa Clara, CA, USA) and neurons were allowed to mature until assay endpoint.

### 2.3. sgRNA guide design

Dual guides were designed using Benchling’s CRISPR Design tool in the second exon of the C9orf72 gene, and optimized for on-target efficiency and minimized for off-target score. Synthetic modified sgRNAs were ordered from Synthego (Redwood City, CA, USA) (sgRNA kit) and resuspended at 100 μM in TE Buffer and stored in the −80°C until use. N*N*N* indicate 2’-O-methyl analogs and 3’-phosphorothioate internucleotide linkages. The sequences are as follows:

Forward guide: U*U*A*ACACAUAUAAUCCGGAA

Reverse guide: C*A*C*ACACUCUAUGAAGUGGG.

### 2.4. Gene editing of iPSCs

Induced pluripotent stem cells were generated from an individual with 2 and 5 copies of the C9orf72 hexanucleotide repeat. To introduce double stranded DNA breaks in exon 2 of C9orf72, iPSCs were electroporated with ribonucleoprotein (RNP) complex of Alt-R SpCas9 Nuclease V3 (Integrated DNA Technologies, Ann Arbor, MI, USA, 1081059) and two different sgRNA guides (Synthego Corporation, Redwood City, CA, USA, sgRNA EZ kit) in a 1–3 molar ratio, respectively, to increase editing efficiency. In short, iPSCs were dissociated to single cells using Accutase for 3 min at in 37°C hypoxic incubator, collected with 10 mL of PBS, then spun at 200 × g for 5 min. The cell pellet was resuspended in PBS and counted using trypan blue on a Countess II (Thermo Fisher Scientific, Waltham, MA, USA). 1e^6 cells were resuspended per electroporation in Neon Buffer R. RNP complexes were formed by incubating Cas9 protein and both sgRNAs for 20 min at room temperature, then adding it to the dissociated cells in Neon Buffer R. A Neon electroporation system (Thermo Fisher Scientific, Waltham, MA, USA) was used to deliver the RNP complexes at two different settings- for TD4 pool: 1100 voltage/20 width/1 pulse, and for TD6 pool: 1,200 voltage/20width/2 pulses.

### 2.5. Clonal isolation

Each pool was sorted into clones by using a Sony SH800 Cell Sorter into 96-well plates pre-filled with 100 μL of NutriStem supplemented with 10 μM ROCK inhibitor at 1 cell/well. Single cells were allowed to grow for 2 weeks in culture. Surviving clones were passaged and reconsolidated into triplicate 96-well plates, one for continued cell culture, DNA isolation, and RNA isolation. Splitting cells was done using EDTA salt solution (Millipore Sigma, Burlington, MA, USA, 03690) into a new plate each time. Cells were banked at 1e6/vial or.5e6/vial in 10% dimethyl sulfoxide (DMSO) (sterile TC grade) and 90% filter-sterilized Knockout Serum Replacement (Thermo Fisher Scientific, Waltham, MA, USA, 10828028).

### 2.6. Genotyping of pools and clones

DNA isolation: 2 days after plating, individual clones were washed with DPBS, before Quick Extract Buffer (Lucigen Corporation Middleton, WI, USA, QE09050) was added. The solution was mixed ten times to lyse the cells prior to long-term storage at −80°C. PCR: 1 μL of DNA from Quick extract was loaded into a PCR using Hot start and the following 10 μM primers: Forward: GATGTCGACTCTTTGCCCAC Reverse: AGGCTCCCAAGAAGAATCCA that amplify a 727 base pair region around the cut site. PCR products were sent out for Sanger sequencing to Genewiz (South Plainfield, NJ, USA). Analysis was performed using Synthego Inference of CRISPR Edits (ICE) Analysis. 2019. v3.0. Synthego; (13 September, 2021) for editing efficiency and determination of knockout sequence.

### 2.7. qPCR for C9orf72 levels

For each cell line, 10,000 cells/well were plated onto Matrigel-coated 96-well plates. 24 h later, total RNA was collected using Cell-to-CT 1-Step TaqMan kit (Invitrogen, Waltham, MA, USA, A2560) following manufacturers specifications. Multiplexed TaqMan was run for *C9orf72* (all variants) (HS00376619_m1, FAM) and GAPDH (Hs03929097_g1, VIC-MGB_PL) on a QuantStudio 7. Biological replicates were run in triplicate with four technical replicates each for all samples and plated using an OT2 (Opentrons Labworks, Inc., Brooklyn, NY, USA). Relative *C9orf72* abundance was quantified using the delta delta CT method. Data was analyzed using GraphPad Prism.

### 2.8. Western blot

Cells were washed with DPBS, spun, and the cell pellet was stored in the −80 for long term storage. Cells pellets were lysed in RIPA (Thermo Fisher Scientific, Waltham, MA, USA, 89900) with Halt Protease inhibitor (Thermo Fisher Scientific, Waltham, MA, USA, 78441) and Dnase (Thermo Fisher Scientific, Waltham, MA, USA, 90083). Cell pellets were vortexed every 5 min on ice for a total of 30 min. Then pipetted up and down with a 32-gauge needle and spun at 12,000 RPM for 15 min at 4°C, and the supernatant was collected. Protein was quantified using a Pierce BCA Protein Assay kit (Thermo Fisher Scientific, Waltham, MA, USA, 23225). A total of 20 μg of protein and 4X Loading Dye Solution (Thermo Fisher Scientific, Waltham, MA, USA, NP0007) were heated to 100°C for 5 min to break down tertiary bonds using 2-mercaptoethanol (Gibco USA, Jenks, OK, USA, 21985–023). Equal amounts of protein were loaded into a NuPAGE 4 to 12% Bis-Tris protein gel (Thermo Fisher Scientific, Waltham, MA, USA, NP0322BOX), and run at 100 V for 120 min in 1X MOPS Buffer (Thermo Fisher Scientific, Waltham, MA, USA, NP0001) on ice. The gel was transferred to PVDF using an iBlot2 at a standardized transfer called P0, which is 20 volts for 1 min, followed by 23 volts for 4 min, then 25 volts for 2 min. Primary antibody against Genetex C9orf72 (GeneTex, Irvine, CA, USA, GTX42591) was used at a 1:400 dilution, and GAPDH (Thermo Fisher Scientific, Waltham, MA, USA, PA1-987) at 1:10,000, separately, in blocking buffer comprised of 5% milk in 1xTBST (Thermo Fisher Scientific, Waltham, MA, USA, 28360) overnight at 4°C. The GeneTex C9orf72 antibody binds to the N-terminus in the longin domain of both the short and long C9orf72 isoforms (Xiao et al., 2016). The next day, three washes were performed using 1xTBST for 10 min each to remove the primary antibody. The membrane was incubated with anti-mouse secondary antibody (LI-COR Biosciences, Lincoln, NE, USA, 926–80010), or anti-rabbit secondary antibody (LI-COR Biosciences, Lincoln, NE, USA, 926–80011), for 1 h at room temperature at a 1:20,000 dilution in blocking buffer. The membrane was then washed three times for 10 min, using 1xTBST. Supersignal West Femto Maximum Sensitivity Substrate (Thermo Fisher Scientific, Waltham, MA, USA, 34095) was added onto the membrane (1:1). The membrane was incubated for 5 min, protected from light at room temperature, and imaged using the Licor C-Digit imager. Densitometric analysis was done using Image J.

### 2.9. Pluripotency staining for iPSCs

Cells were rinsed once with DPBS and fixed with 4% PFA for 10 min. PFA was removed with three DPBS rinses, prior to blocking with 3% donkey serum at 4°C overnight. The next day, primary antibodies for Oct3/4, Nanog, Tra-1–60, and Tra-1–81 were added and incubated overnight at 4°C. On the 3 day, primary antibody solutions were removed with three DPBS rinses, before AF488 or AF594 -conjugated secondary antibodies were added for 1 h at room temperature. After rinsing off the secondary antibody solutions, cells were stained with 1 μg/mL DAPI (Thermo Fisher Scientific, Waltham, MA, USA, 62248) for 10 min, before imaging on a Cytation3 automated microscope from BioTek with a 4X objective.

### 2.10. CellTracker Green CMFDA and Hoescht 333342 staining and imaging

Motor neurons progenitors were dissociated and plated on borate/poly-ornithine coated 384-well plates in laminin supplemented NDM. After 16 h, neurons were stained with 5 μM CellTracker Green and Hoechst 333342 (Thermo Fisher Scientific, Waltham, MA, USA, H3570) following the manufacturers protocol. In short, cells were washed once with NDM, then stained with 10 μM CellTracker Green CMFDA and 1.2 nM Hoechst 333342 for 30 min at 37°C. After incubation, cells were rinsed twice with NDM without phenol-red prior to imaging on a Cytation3 microscope using a 20X objective.

### 2.11. Storage and dilution of polyGR_15_

Synthesized polyGR_15_ (95.18% purity by HPLC, MW 3216.57 g/mol) (GenicBio Limited, Kowloon, Hong Kong SAR, China) was purchased as lyophilized powder and resuspended in DMSO at 10 mM. Aliquots were stored at −80°C until use.

### 2.12. Storage and dilution of PRMT inhibitor MS023 and inactive analog MS094

Protein arginine methyltransferase inhibitors MS023 (MedChem Express, HY-19615, Monmouth Junction, NJ, USA) and MS094 (Millipore Sigma, SML2548, Burlington, MA, USA) were both resuspended in DMSO at 10 mM and stored in the −80°C until use.

### 2.13. Preparation and dosing of polyGR_15_ and PRMT inhibitor

Separately, both polyGR_15_ and MS023, and MS094 were serially diluted into (for DMEM/F12) Gibco (Jenks, OK, USA) before combining in a 96-well source plate at a 2X concentration. 12 days post-plated MNs in 384-well plates were rinsed with complete NDM, and treated with the compound source plate at a 1:1 ratio. MNs were incubated for 24 or 48 h prior to assessing viability by CellTiter-Blue (Promega Corporation, Madison, WI, USA, G8081) fluorescence or live-cell imaging. Doses for exogenous application of polyGR_15_ were chosen based on previously established uptake profiles of synthetic DPR constructs in various cell models (Kwon et al., 2014; Kanekura et al., 2018; Gill et al., 2019; Lafarga et al., 2021).

### 2.14. CellTiter-Blue viability measurements

CellTiter-Blue (Promega Corporation, Madison, WI, USA, G8081) was thawed for 30 min in a water bath at 37°C. For cells in a 96-well format, 20 μL of undiluted CellTiter-Blue was added, and cells were incubated at 37°C for 2.5 h, followed by a top-well fluorescence read (Ex. 560, Em. 590) using a Cytation3. For 384-well plates, CellTiter-Blue was diluted 1:5 into DPBS, then plates were aspirated leaving 22 μL/well, followed by 10 μL diluted CTB addition and a 2.5 h incubation at 37°C. Background subtraction was performed across each plate using wells that did not contain cells.

### 2.15. Neurite outgrowth analysis

Neurite outgrowth was quantified using PerkinElmer Columbus software. Images were pre-processed with a sliding parabola filter (curvature 100) for the green channel. Nuclei were identified using Hoechst, and cytoplasm were segmented using the green channel. Neurites were identified using CSIRO neurite analysis 2.

### 2.16. C9orf72-HRE genotyping

Fibroblast genomic DNA was isolated using the Zymo Quick-DNA Miniprep Plus Kit (Zymo Research, Irvine, CA, USA, D4069) following manufacturers recommended protocol. Amplicon length analysis and RP-PCR were performed following (DeJesus-Hernandez et al., 2011), with slight modifications.

#### 2.16.1. Amplicon length analysis

A total of 20 μL PCR reactions were composed of the following: 1 μL of 100 ng/μL genomic DNA, 1 μL 10X PCR Buffer (Qiagen, Germantown, MD, USA, 201203), 0.5 μL of 5 mM dATP, dCTP, dTTP mix (Invitrogen, Waltham, MA, USA, 10297117), 0.5 μL of 5 mM daeza-dGTP (New England Biolabs, Ipswich, MA, USA, N0445L), 1 μL of 10 μM forward and reverse primers (forward: Fam-CAAGGAGGGAAACAACCGCAGCC, reverse: GCAGGCACCGCAACCGCAG), 0.5 μL of 100% DMSO (Sigma-Aldrich, Burlington, MA, USA, D2650), 2 μL of 5M betaine (Sigma-Aldrich, Burlington, MA, USA, B0300-1VL), 0.1 μL Taq polymerase (Qiagen, Germantown, MD, USA, 201205), 9 μL of water. The following thermocycler protocol was performed: 98°C for 5 min, 11 cycles of the following: 97°C for 0:30 min, 55°C for 0:30 min (−1°C/cycle), 68°C for 1:30 min, 24 cycles of the following: 97°C for 0:30 min, 55°C for 0:30 min, 68°C for 1:30 min, a final extension at 68°C for 10 min, followed by a 4°C hold. The resulting PCR product was diluted 1 to 30 with water, and 2 μL was sent to the Georgia Genomics Facility for fragment analysis on an Applied Biosystems 3730xl DNA analyzer. ROX500 and formamide were added to the samples.

#### 2.16.2. Repeat-primed PCR analysis (RP-PCR)

A total of 40 μL PCR reactions were composed of the following: 1 μL of 10 ng/μL genomic DNA, 2 μL 10X PCR Buffer (Qiagen, Germantown, MD, USA, 201203), 1 μL of 5 mM dATP, dCTP, dTTP mix (Invitrogen, Waltham, MA, USA, 10297117), 0.5 μL of 5 mM daeza-dGTP (New England Biolabs, Ipswich, MA, USA, N0445L), 2 μL of 10 μM primer mix (forward: FAM-TGTAAAACGACGGCCAGTCAAGGAGGGAAACAACCGCAG CC, anchor: CAGGAAACAGCTATGACC, reverse: CAGGAAA CAGCTATGACCGGGCCCGCCCCGACCACGCCCCGGCCCC GGCCCCGG), 1 μL of 100% DMSO (Sigma-Aldrich, Burlington, MA, USA, D2650), 4 μL of 5M betaine (Sigma-Aldrich, Burlington, MA, USA, B0300-1VL), 0.2 μL Taq polymerase (Qiagen, Germantown, MD, USA, 201205), 19 μL of water. The following thermocycler protocol was performed: 96.5°C for 12:30 min, 16 cycles of the following: 95°C for 1:00 min, 70°C for 1:00 min (−0.5°C/cycle), 72°C for 3:00 min, six cycles of the following: 95°C for 1:00 min, 61°C for 1:00 min (−1°C/cycle), 72°C for 3:00 min, 35 cycles of the following: 95°C for 1:00 min, 55°C for 1:00 min, 72°C for 3:00 min, a final extension at 72°C for 10 min, followed by a 4°C hold. The resulting PCR product was diluted 1 to 30 with water, and 2 μL was sent to the Georgia Genomics Facility for fragment analysis on an Applied Biosystems 3730xl DNA analyzer. ROX1000 and formamide were added to the samples. For both ALA and RP-PCR, trace files were analyzed using GeneMarker software (Softgenetics).

### 2.17. Longitudinal live-cell imaging

VACHT-tdTomato MNs were plated at a density of 700 cells per well in a 384-well. 13 days post-plating, an initial imaging timepoint of the entire plate was taken at 24 h prior to treatment with polyGR_15_ and MS023. Following 24 h post-treatment, VACHT-tdTomato cells were imaged every 6 h starting using a Cytation 3 microscope with a 4x objective. Four images were taken to capture the whole well, and the images were stitched into a composite image. Neuronal somas were quantified and normalized to the to the same well’s previous time point.

### 2.18. Statistical analysis

Statistical analyses were performed using GraphPad Prism v.9.4.1, Microsoft Excel, and TIBCO Spotfire v11.4.3. Statistical tests included one-way and two-way ANOVAs with Dunnett’s multiple comparisons test, and four parameter logistical regression models to calculate IC50s. Experiments were performed in technical quadruplicates, with biological replicate *n* values greater than three or four.

## 3. Results

**FIGURE 1:**
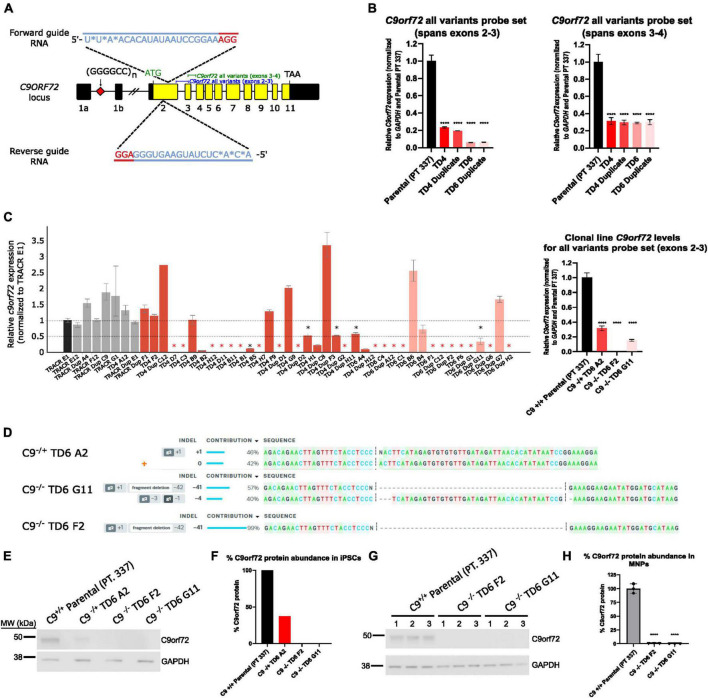
Generation of hemizygous and homozygous *C9orf72* knockout induced pluripotent stem cells (iPSCs). **(A)** Schematic map of the C9orf72 genomic region and the relative binding positions of forward and reverse sgRNA guides used to introduce an INDEL mutation in exon 2, and TaqMan probe sets used to quantify expression. Diagram modified from Shi et al. (2018). **(B)** Expression of *C9orf72* by quantitative reverse transcription polymerase chain reaction (*qRT-PCR*) in pooled cell populations following Cas9:sgRNA RNP electroporation. Values were normalized to parental cells. ****Denotes *p* < 0.01 (comparing each pooled cell line to the parental pool by one-way ANOVA analysis of variance followed by Dunnett’s test). Data are presented as mean values ± standard deviations (error bars). **(C)** Expression of *C9orf72* by qRT-PCR in a large screen of isolated clonal lines, as well as in a smaller subset of clonal lines that were used in this study. Black asterisk denotes potential heterozygous KO clones, and red asterisk denotes potential homozygous KO clones. **(D)** ICE analysis of Sanger sequencing of edited exon 2 region. **(E)** *C9orf72* protein levels shown by Western blotting for iPSC clonal lines. **(F)** Relative quantification of the C9orf72 protein levels shown in **(E)**. Data are presented as mean values ± standard deviations (error bars). **(G)** *C9orf72* protein levels shown by Western blotting for motor neuron progenitor clonal lines run in triplicate. **(H)** Relative quantification of the *C9orf72* protein levels shown in **(G)**. *C9*^–^*^/^*^–^ TD6 G11 lane three only contains 10 μg of the total protein (50%) due to insufficient volume.

A dual guide approach targeting exon 2 was used to successfully knockdown *C9orf72* expression in pooled cells (Figure 1A). qPCR showed successful knockdown in *C9orf72* mRNA levels with an 80–90% reduction in four pools (TD4, TD4 Duplicate, TD6, TD6 Duplicate) using an exon 2–3 *C9orf72* probe set, which amplifies all three splice isoforms (Figure 1B). A different total *C9orf72* probe set, which spans exons 3–4, showed a lower reduction (∼70%) in *C9orf72* expression (Figure 1B). After isolation of isogenic clones, a qPCR revealed that the levels of detectable *C9orf72* ranged from 0 to 50% compared to Tracr E1 edited control cells in many clones (Figure 1C). From this large screen of clones, a few candidate clones were selected (not all ∼150 clones shown). One hemizygous clone and two homozygous knockout clones were selected for further analysis. *C9orf72* expression analyzed by qPCR showed that compared to the parental line (Pt. 337), total *C9orf72* was reduced by 70% in the hemizygous clone (TD6 A2), and 100 and 85% in the two homozygous clones, TD6 F2 and TD6 G11, respectively (Figure 1C). Expression of *C9orf72* splice variant 1 and 3 were dramatically lower than total C*9orf72*, suggesting that variant 2 is the most abundant splice isoform (Supplementary Figure 1). For hemizygous clone TD6 A2, ICE analysis following Sanger sequencing around the deletion showed edits on 1 allele, with 1 nucleotide insertion contributing 46%, and the wild-type allele contributing 42% (Figure 1D). The homozygous clone, TD6 G11 had a 41bp deletion contributing 57% and 4bp deletion at 40% and clone TD6 F2, had a 41bp deletion contributing 99% (Figure 1D). Western blot analysis showed no expression of the C9orf72 protein in the iPSC homozygous knockout clones (TD6 F2 and G11) and an expression lower than 50% in the iPSC hemizygous clone (TD6 A2) (Figures 1E, F). Western blot analysis using motor neuron progenitors at 44 days post-differentiation showed no C9orf72 protein expression in both homozygous knockout clones (TD6 F2 and G11) (Figures 1G, H). Full uncropped blots for Figures 1E, F are shown in Supplementary Figure 6. Following editing and clonal isolation, all cell lines retained expression of pluripotency markers Oct 3/4, Tra-1–60, Tra-1–81, and Nanog (Supplementary Figure 2).

**FIGURE 2:**
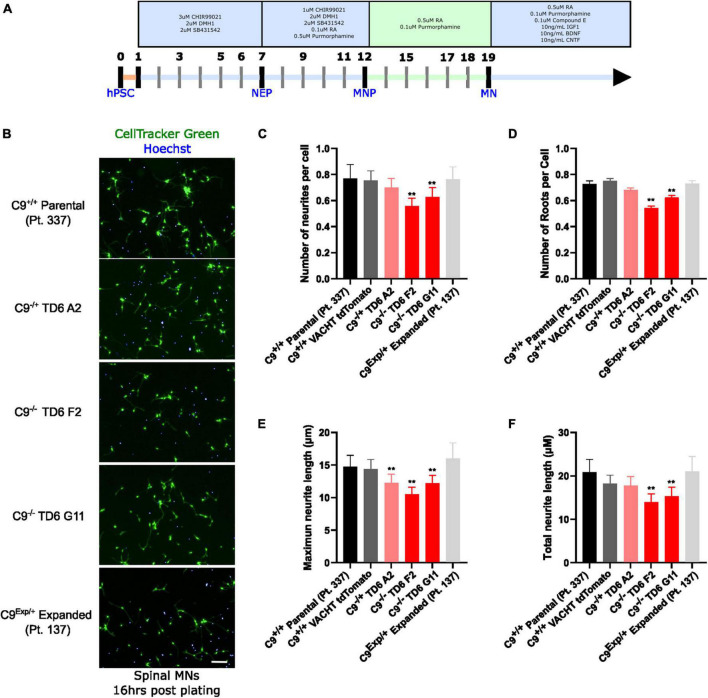
Reduced C9orf72 reduces neurite outgrowth. **(A)** Schematic timeline for spinal motor neuron differentiation. Adapted from Du et al. (2015) **(B)** live-cell imaging of spinal motor neurons stained with Cell Tracker Green and Hoechst 16 h after plating. Scale bar = 100 μm. **(C)** Quantification of the average number of neurite extremities per cell. **(D)** Quantification of the number of neurite roots per cell. **(E)** Quantification of the maximum neurite length per cell. **(F)** Quantification of total neurite length per cell. **Denotes *p* < 0.01 [comparing each cell line to C9^(+/+)^ parental neurons by one-way ANOVA analysis of variance followed by Dunnett’s test]. Data are presented as mean values ± standard deviations (error bars).

**FIGURE 3:**
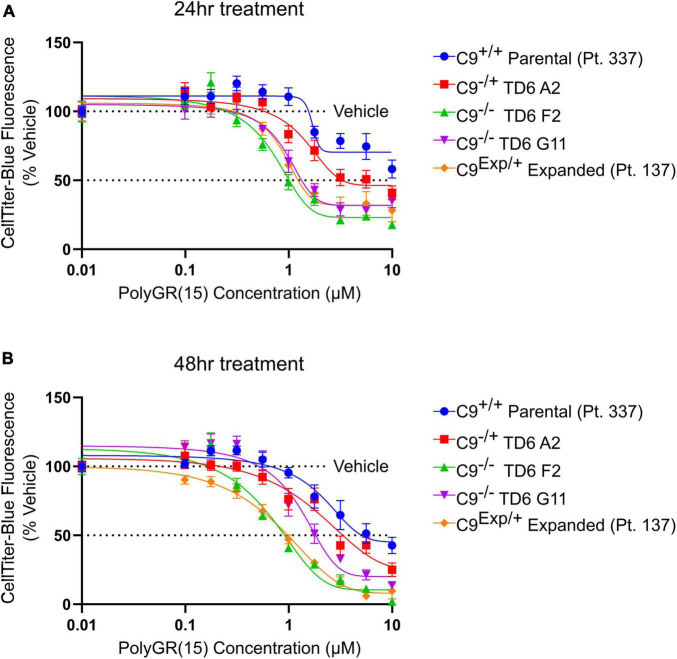
Reduced C9orf72 expression exacerbates polyGR_15_ toxicity in induced pluripotent stem cell (iPSC)-derived motor neurons. **(A)** Neuronal survival 24 h after exogenous treatment with polyGR_15_ ranging from 10 nM to 10 μM or 0.1% DMSO control. Values were normalized to vehicle treated wells for each cell line. **(B)** Neuronal survival 48 h after exogenous treatment with polyGR_15_ or 0.1% DMSO control. Values were normalized to vehicle treated wells for each cell line. Data are presented as mean values ± standard deviations (error bars).

In addition to the isogenic knockout clones, a wild-type NCRM1-TDT VACHT-ICRE or VACHT-tdTomato line was used (Garcia-Diaz et al., 2020). This line was selected due to its expression of the fluorescent tdTomato protein when the VACHT promotor is active, allowing longitudinal tracking of neuronal survival. An iPSC line from a patient with a C9orf72-expansion (Pt. 137) was also included. C9-HRE genotyping was performed using amplicon length analysis (ALA) and repeat-primed PCR (RP-PCR) (Supplementary Figure 3). All lines were then differentiated into spinal motor neurons using a chemically defined protocol (Du et al., 2015) that mimics normal embryonic development (Figure 2A). In C9^+/+^ motor neuron progenitors (MNPs), expression of C*9orf72* increased by 5-fold compared to iPSCs (Supplementary Figure 1F). The levels of total C*9orf72* transcript was reduced ∼100–80% in the homozygous KO lines compared to controls in MNPs. No significant difference in the ability to generate Hb9^+^ spinal motor neurons was observed between lines (Supplementary Figure 4). Neurons were stained with CellTracker Green 16 h after plating to assess early neuronal morphology (Figure 2B). The number of neurites per cell, number of roots per cell, maximum neurite length and total neurite length were calculated (Figures 2C–F). Compared to the parental wild-type cell line, homozygous knockout lines had significantly less neurite outgrowth. The hemizygous line had slightly less outgrowth compared to control lines, however, only differences in maximum neurite length were significant. The expanded patient line showed similar levels to that of our control Pt. 337 line for all of these neurite statistics across all quantifications. This may be due to differences in genetic background between the genome-edited neurons and the expanded patient line. After generating sMNs from each line, we applied a concentration range of polyGR_15_ and assessed cell viability after 24 and 48 h of exposure. Cell viability was measured using the resazurin-based fluorescent dye, CellTiter-Blue. We found that the parental Pt. 337 control neurons with intact C9orf72 were not as sensitive to polyGR_15_ compared to the hemizygous knockout line containing one copy of C9orf72, and compared to the homozygous knockout lines, which were the most sensitive to polyGR_15_ (Figures 3A, B). Statistical analysis is shown in Table 1. Interestingly, the expanded patient with one copy of C9orf72 intact and one copy of C9orf72 mutated performed the same in response to polyGR_15_ challenge as the homozygous knockout lines with no intact copies of C9orf72. The inverse correlation between C9orf72 expression and sensitivity to polyGR_15_ was present at both 24–48 h challenge endpoints.

**TABLE 1:** Statistical testing of data in Figure 3 indicates significant differences C9^(+/+)^ parental (Pt. 337) and edited lines following exposure to PolyGR_15_^1^.

Endpoint	Cell line vs. C9^(+/+)^ parental	10 μM	5.618 μM	3.156 μM	1.773 μM	0.996 μM	0.56 μM	0.314 μM	0.177 μM	0.099 μM	Vehicle	IC50	R^2^
24 h	C9^(+/+)^ parental	NA	NA	NA	NA	NA	NA	NA	NA	NA	NA	49.07	0.54
24 h	C9^(–/+)^ TD6 A2	NS	*	**	NS	**	NS	Ns	NS	NS	NS	20.04	0.75
24 h	C9^(–/–)^ TD6 F2	****	****	****	****	****	****	**	NS	NS	NS	4.225	0.85
24 h	C9^(–/–)^ TD6 G11	*	****	****	****	****	**	Ns	NS	NS	NS	8.411	0.80
24 h	C9^(–/+)^ Expanded Pt. 137	***	****	****	****	****	**	Ns	NS	NS	NS	7.394	0.80
48 h	C9^(+/+)^ parental	NA	NA	NA	NA	NA	NA	NA	NA	NA	NA	46.53	0.67
48 h	C9^(–/+)^ TD6 A2	NS	NS	*	NS	*	NS	NS	NS	NS	NS	5.027	0.72
48 h	C9^(–/–)^ TD6 F2	****	****	****	****	****	****	**	NS	NS	NS	2.675	0.90
48 h	C9^(–/–)^ TD6 G11	***	***	****	**	**	NS	Ns	NS	NS	NS	12.86	0.85
48 h	C9^(–/+)^ Expanded Pt. 137	****	****	****	****	****	****	***	**	NS	NS	2.227	0.91

^1^*Denotes *p* < 0.05, **denotes *p* < 0.01, ***denotes *p* < 0.001, and ****denotes *p* < 0.0001 comparing changes in neuronal survival at each concentration compared to C9^(+/+)^ parental by two-way ANOVA analysis of variance followed by Dunnett’s test. IC50s were calculated using a four-parameter logistical regression model.

**FIGURE 4:**
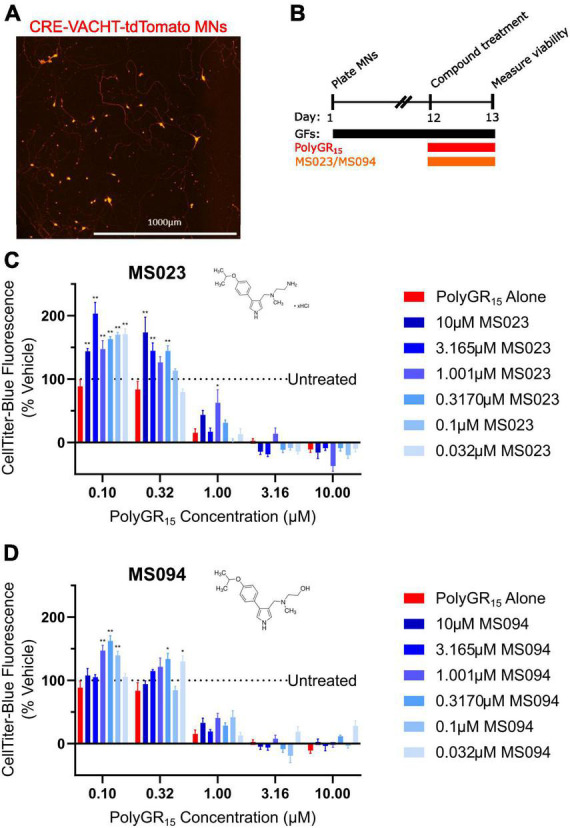
Protein arginine methyltransferase inhibition improves induced pluripotent stem cell (iPSC)-motor neuron (MN) survival in response to polyGR_15_ challenge. **(A)** Representative image of tdTomato^+^ CRE-VACHT-tdTomato control motor neurons employed in polyGR_15_–PRMT inhibitor cross-titration. **(B)** Timeline for cross-titration experiment. Neurons were differentiated to spinal motor neurons, and on day 1, dissociated and plated into 384-well plates. After 12 days, neurons were treated with polyGR_15_ concentrations ranging from 100 nM to 10 μM, and co-treated with MS023 or MS094 concentrations ranging from 32 nM to 10 μM. Cell survival was measured 24 or 48 h after treatment. **(C)** Neuronal survival following polyGR_15_ co-treated with MS023 normalized to vehicle treated wells (0.2% DMSO). **(D)** Neuronal survival following polyGR_15_ co-treated with MS023 normalized to vehicle treated wells (0.2% DMSO). *Denotes *p* < 0.05, and **denotes *p* < 0.01 (comparing MS023 or MS094 co-treated groups to polyGR_15_ alone by one-way ANOVA analysis of variance followed by Dunnett’s test). Data are presented as mean values ± standard deviations (error bars).

**FIGURE 5:**
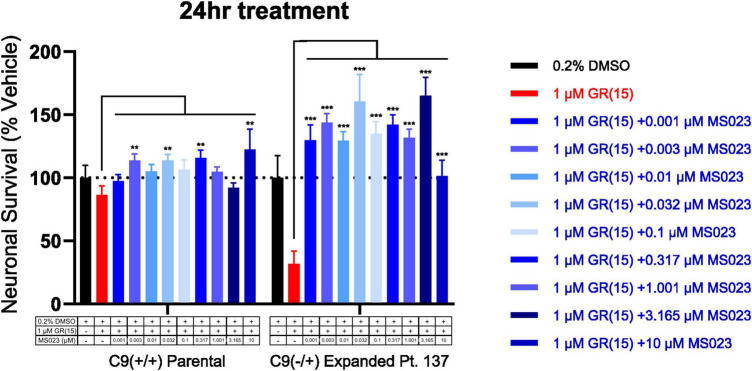
Protein arginine methyltransferase inhibition rescues polyGR_15_-induced toxicity in C9orf72-expanded motor neurons. Survival of spinal motor neurons 24 days post-plating following co-treatment with 1 μM polyGR_15_ and concentrations of MS023 ranging from 1 nM to 10 μM. **Denotes *p* < 0.01, and ***denotes *p* < 0.001 (comparing MS023 co-treated groups to polyGR_15_ alone by two-way ANOVA analysis of variance followed by Dunnett’s test). Data are presented as mean values ± standard deviations (error bars).

Previous work has shown that inhibition of Type I PRMTs reduces toxicity of arginine-rich DPRs in NSC-34 and primary mouse spinal motor neurons (Premasiri et al., 2020). To test if these results would replicate in iPSC-derived sMNs, the VACHT-tdTomato reporter line was used (Figure 4A). Following terminal differentiation, day 12 human sMNs were co-treated with polyGR_15_ and either Type I PRMT inhibitor MS023, or its inactive analog MS094 before measuring viability with CellTiter-Blue (Figure 4B). Addition of MS023 was able to rescue neuronal viability against 0.1–1.0 μM polyGR_15_ (Figure 4C). Surprisingly, the inactive analog MS094 was able to increase survival against 0.1–0.32 μM polyGR_15_ (Figure 4D). To assess cell viability using a different strategy, we employed longitudinal live-cell imaging to measure the number of tdTomato^+^ MNs every 6 h following application of 1–5.62 μM polyGR_15_ alone, or with co-treated with MS023 (Supplementary Figures 5A–D). Here, a concentration-dependent rescue of neuronal survival was seen with 0.1–1.0 μM MS023 against polyGR_15_ induced-toxicity. The preservation of intact neurites was seen 84 h post-polyGR_15_ when 1 μM MS023 was present (Supplementary Figure 5A). Lastly, we sought to determine if type I PRMT inhibition was beneficial in C9orf72-expanded MNs. Day 24 MNs were co-treated with 1 μM polyGR_15_ and 1 nM–10 μM MS023 for 24 h prior to cell survival assessment by CellTiter-Blue fluorescence (Figure 5). The increased sensitivity to 1 μM polyGR_15_ was again seen in the C9-expanded MNs. MS023 abrogated polyGR_15_ toxicity seen in the C9-expanded cells.

## 4. Discussion

A decade following the association of *C9orf72* repeat expansion mutations in many cases of familial and sporadic ALS and FTD there have been tremendous strides in elucidating processes that may be implicated in these diseases (Balendra and Isaacs, 2018; Masrori and Van Damme, 2020). *C9orf72* mutation-mediated neurodegeneration has proven to be complex, both biologically and clinically. Prominent hypotheses implicate both gain-of-function and loss-of-function mechanisms that lead to neuronal cell death. Thusly, treatment approaches should aim to address both, as few research studies have explored the additive toxic effects of both. Approaches to treat the *C9orf72* HRE have mostly aimed at reducing toxic gain-of-function– with multiple antisense oligonucleotide therapeutics in clinical development now (Liu et al., 2021; Tran et al., 2022) or in recent months (Lagier-Tourenne et al., 2013). Biogen’s IONIS-C9Rx is an ASO targeting the *C9orf72* open reading frame mRNA, which is aimed at reducing the DPRs translated from the expansion. Similarly, Wave Life Science’s WVE-3972–01 is a sterically pure ASO targeting downregulation of DPRs and pathogenic *C9orf72-*derived RNA transcripts and claims to have kept intact the healthy C9orf72 transcriptional V2 variants (Liu et al., 2022). These treatments aim to address downstream effects within the cell. However, it is important to consider a therapy that does not reduce levels of wild-type C9orf72. The association between increased levels of transcript variant 1 in the frontal cortex and cerebellum with longer survival in *C9orf72* expansion carriers should caution against therapies that reduce C9orf72 levels (van Blitterswijk et al., 2015). Several small molecule strategies have also been explored for treatment of *C9orf72-*linked neurodegeneration. Metformin, an FDA-approved drug for the treatment of type 2 diabetes, is being repurposed as a small molecule approach to reduce production of toxic *C9orf72* non-canonical translation products (Zu et al., 2020). A repositioned small molecule drug, TPN101, is being developed for C9orf72 ALS (NCT04993755) with an emphasis on its potential to reduce central nervous system inflammation (Zampatti et al., 2022). One challenge when developing these small molecule treatment approaches is careful consideration to avoid lowering levels of endogenous C9orf72 protein when targeting sequences adjacent to the hexanucleotide repeat, aiming to instead target repeat-containing RNAs (Zhu et al., 2020; Bush et al., 2022). One small molecule developed with dual function was able to bind the expansion and recruit an endonuclease to remove the expansion from the RNA without effecting the levels of C9orf72 protein (Bush et al., 2022).

We show that human iPSC-derived MNs lacking one or both of the C9orf72 alleles are more susceptible to polyGR_15_ repeat toxicity. Additionally, we demonstrate that iPSC-derived motor neurons from a person harboring one C9orf72-expanded allele are similarly sensitive to polyGR_15_ toxicity as cells completely lacking C9orf72 alleles. These findings may help elucidate the biological cascade of events that result in C9orf72 mutation-mediated neuronal cell death. One of the earliest observations about C9orf72 neurodegeneration was that people harboring repeat expansion mutations manifest reduced expression of *C9orf72* transcript variants 1 and 2 (DeJesus-Hernandez et al., 2011; van Blitterswijk et al., 2015). This suggested the possibility of toxic loss-of-function playing a role in *C9orf72-*linked neurodegeneration. Our results are consistent with the idea that production of toxic DPRs in the context of reduced C9orf72 in people with repeat expansions would be more likely to result in neuronal cell death than the same exposure to toxic DPR in the context of normal C9orf72 expression levels. Interestingly, the fact that toxic DPR exposure was equivalently toxic to cells from a person with one expanded *C9orf72* allele and cells with a *C9orf72* double knockout, suggests that additional gain-of-function mechanisms might be playing a role as well. These might include toxic DPRs translated from the expanded repeat allele or toxic repeat expanded sense or antisense RNAs. In future studies, we will have to consider getting a baseline for the amount of endogenous polyGR present in each sample before addition of exogenous polyGR_15_ in order to determine background toxicity caused by endogenous polyGR.

Equally important in this study is the observation that inhibition of asymmetric arginine dimethylation by Type I PRMT inhibitor MS023 was protective against polyGR_15_ induced toxicity in iPSC-derived sMNs. We have previously observed that MS023 can completely abrogate toxicity resulting from polyGR_15_ and polyPR_15_ challenge in NSC-34 cells, a motor neuron-like cell line (Premasiri et al., 2020). Further, showing this phenomenon is present in iPSC-derived MNs from an expanded patient using polyGR_15_ implicates PRMTs in ALS disease pathology, and shows that it holds promise at elucidating further study into the mechanism of action of this rescue phenotype. This could shed light on how inhibiting PRMTs could lead to rescue of patient cells from toxicity from polyGR_15_. Our observation that MS023 rescues cell viability above vehicle-treated cells when measured using CellTiter-Blue is worth further investigation. Reduction of resazurin to resorufin present in CellTiter-Blue by the mitochondrial respiratory chain in live cells suggests metabolic activity is enhanced following MS023 treatment in the presence of polyGR_15_. It has been shown that PRMT inhibition by MS023 treatment results in global transcriptomic and proteomic alterations (Maron et al., 2021). The fact that MS023 also rescued neuron survival and preserved neurite integrity agrees with previous NSC-34, where PRMT inhibition rescued both metabolic activity and LDH release following DRP challenge (Premasiri et al., 2020). Further work to better understand the mechanism of rescue is critical to designing more selective PRMT inhibitors. Modulation of the levels of each Type I PRMT, through knockdown or knockout, will help shed light on which enzymes should be targeted.

We have previously described evidence supporting roles of protein arginine methylation in relation to cell stress responses such as splicing, chromatin remodeling, and stress granule dynamics in C9orf72 cell-based gain-of-function models (Gill et al., 2021). The current studies reveal a likely interaction between native C9orf72 protein and PRMTs in regulating these pathways in C9orf72-mutation-mediated neurodegeneration. Recent studies have highlighted the importance of stress granule formation and requisite liquid-liquid phase separation as modulators of C9orf72 mutation-mediated cytotoxicity in cell-based models (Liu et al., 2023; Millar et al., 2023). For example, C9orf72 has been demonstrated to be a regulator of the integrated stress response (ISR), facilitating stimulation of the unfolded protein response (UPR), inhibition of stress granule formation, and LLPS through interaction with eIF2a (Zheng et al., 2022). Accordingly, loss of C9orf72 results in enhanced stress granule formation and non-canonical translation of toxic DPRs in a feed-forward mechanism (Cheng et al., 2018). Additionally, protein arginine methylation of stress granule-associated proteins regulates phase separation, stress granule dynamics, and translation in stressed cells. Indeed, the type III PRMT, PRMT7, methylates eIF2a in a manner required for eIF2a-dependent stress granule formation (Haghandish et al., 2019). Additionally, arginine methylation of RGG/RG motifs within stress granule proteins can limit their interactions with RNA, poly (ADP-ribose), and other stress granule components (Tsang et al., 2019). Notably, methylation of key stress granule protein G3BP1 by PRMT1 prevents large stress granule formation (Tsai et al., 2016), and asymmetric dimethylation of several RNA binding proteins results in their recruitment to stress granules (Xie and Denman, 2011). Multiple described roles for PRMTs driving stress granule assembly and composition indicate that PRMT activity might influence the ISR in a manner protective to C9orf72-deficient cells.

Further, PRMT activity has been shown to modulate autophagy (Choi et al., 2019). In the case of simultaneous C9orf72 loss-of-function and DPR gain-of-function challenge modeled in the current experiments, pharmacological inhibition of Type I PRMT activity in C9orf72-deficient cells might increase autophagic clearance of DPRs by way of compensatory Type II PRMT substrate scavenging (Dhar et al., 2013; Stouth et al., 2017). Symmetric dimethylation of stress granule proteins by PRMT5 has been shown to drive the association of C9orf72 and p62 to the stress granule as a complex that works to eliminate stress granules by autophagy (Chitiprolu et al., 2018). In the present study, application of the Type I PRMT inhibitor MS023 concurrent with polyGR challenge most significantly rescued survival of iPSC-derived motor neurons harboring the C9orf72 repeat expansion mutation (Figure 5). Future studies should determine whether Type I PRMT inhibition enhances symmetric dimethylation of stress granule proteins, and subsequently drives more native C9orf72 protein to associate with p62 to promote protective autophagy of either accumulated DPRs or other deleterious inclusions. Such a mechanism could act to ameliorate reduced impaired autophagy observed in C9orf72 *in vitro* knockdown models (Sellier et al., 2016; Webster et al., 2016) and subsequent increases in DPR toxicity (Boivin et al., 2020; Zhu et al., 2020).

Other vital cellular mechanisms are co-regulated by C9orf72 protein and PRMT activity. One recent publication found that C9orf72 is involved in recruiting DNA repair enzymes to double-stranded breaks when localized to the nucleus, and that repair of double-stranded DNA breaks following polyGR overexpression is reduced in C9orf72-deficient cells (He et al., 2022). PRMTs have a vast set of roles in the DNA damage response that vary by PRMT enzyme and arginine methylation type (Lorton and Shechter, 2019; Urulangodi and Mohanty, 2020; Brobbey et al., 2022). Modulation of PRMT activity in the absence of C9orf72-mediated DNA damage repair may act to mitigate DNA damage response deficits in ALS loss-of-function and gain of function models. Recently, one study illustrated that loss of C9orf72 disrupts the Ran-GTPase gradient and nucleocytoplasmic transport in both *in vitro* and *in vivo* models (McGoldrick et al., 2023). Further, this study illustrated that abundance of importin β-1 granules is increased in states of C9orf72-deficiency. PRMT activity also modulates the Ran-GTPase gradient and nucleocytoplasmic transport. Namely, chromatin-bound RCC1 activates Ran-GTPase to initiate nucleocytoplasmic transport, and localization of RCC1 to chromatin requires methylation of RCC1 by PRMTs (Huang et al., 2021). Additionally, PRMT-mediated arginine methylation at nuclear transport domains (NTDs) and nuclear localization sequences (NLSs) regulates nucleocytoplasmic transport through interaction with importin proteins, including importin β (Smith et al., 2004). These significant overlaps in regulatory roles of PRMTs and C9orf72 warrant further investigation into whether modulation of PRMT behavior in C9orf72 mutation-mediated neurodegeneration might influence C9orf72 loss-of-function and gain-of-function consequences.

## Data availability statement

The original contributions presented in this study are included in the article/Supplementary material, further inquiries can be directed to the corresponding author.

## Author contributions

TD and KD contributed to conception, design of the study, performed the statistical analysis and made figures. AG provided study design guidance and reagents based on previous data for formulations and dosages. KD, AG, FV, and TD wrote the first draft of the manuscript, introduction, and discussion. All authors contributed to manuscript revision, read, and approved the submitted version.

## References

[B1] Amick J. Roczniak-Ferguson A. Ferguson S. M. (2016). C9orf72 binds SMCR8, localizes to lysosomes, and regulates mTORC1 signaling. *Mol. Biol. Cell.* 27 3040–3051. 10.1091/mbc.e16-01-0003 27559131 PMC5063613

[B2] Aoki Y. Manzano R. Lee Y. Dafinca R. Aoki M. Douglas A. G. L. (2017). C9orf72 and RAB7L1 regulate vesicle trafficking in amyotrophic lateral sclerosis and frontotemporal dementia. *Brain* 140 887–897. 10.1093/brain/awx024 28334866

[B3] Ash P. E. A. Bieniek K. F. Gendron T. F. Caulfield T. Lin W. L. DeJesus-Hernandez M. (2013). Unconventional translation of C9ORF72 GGGGCC expansion generates insoluble polypeptides specific to c9FTD/ALS. *Neuron* 77 639–646. 10.1016/j.neuron.2013.02.004 23415312 PMC3593233

[B4] Balendra R. Isaacs A. M. (2018). C9orf72-mediated ALS and FTD: Multiple pathways to disease. *Nat. Rev. Neurol.* 14 544–558. 10.1038/s41582-018-0047-2 30120348 PMC6417666

[B5] Blanchet F. Cardona A. Letimier F. A. Hershfield M. S. Acuto O. (2005). CD28 costimulatory signal induces protein arginine methylation in T cells. *J. Exp. Med.* 202 371–377. 10.1084/jem.20050176 16061726 PMC2213083

[B6] Boivin M. Pfister V. Gaucherot A. Ruffenach F. Negroni L. Sellier C. (2020). Reduced autophagy upon C9ORF72 loss synergizes with dipeptide repeat protein toxicity in G4C2 repeat expansion disorders. *EMBO J.* 39:e100574. 10.15252/embj.2018100574 31930538 PMC7024836

[B7] Braems E. Swinnen B. Van Den Bosch L. (2020). C9orf72 loss-of-function: A trivial, stand-alone or additive mechanism in C9 ALS/FTD? *Acta Neuropathol.* 140 625–643. 10.1007/s00401-020-02214-x 32876811 PMC7547039

[B8] Brobbey C. Liu L. Yin S. Gan W. (2022). The role of protein arginine methyltransferases in DNA damage response. *IJMS* 23:9780. 10.3390/ijms23179780 36077176 PMC9456308

[B9] Bush J. A. Meyer S. M. Fuerst R. Tong Y. Li Y. Benhamou R. I. (2022). A blood–brain penetrant RNA-targeted small molecule triggers elimination of r(G _4_ C _2_) ^exp^ in c9ALS/FTD via the nuclear RNA exosome. *Proc. Natl. Acad. Sci. U. S. A.* 119:e2210532119. 10.1073/pnas.2210532119 36409902 PMC9860304

[B10] Cheng W. Wang S. Mestre A. A. Fu C. Makarem A. Xian F. (2018). C9ORF72 GGGGCC repeat-associated non-AUG translation is upregulated by stress through eIF2α phosphorylation. *Nat. Commun.* 9:51. 10.1038/s41467-017-02495-z 29302060 PMC5754368

[B11] Chitiprolu M. Jagow C. Tremblay V. Bondy-Chorney E. Paris G. Savard A. (2018). A complex of C9ORF72 and p62 uses arginine methylation to eliminate stress granules by autophagy. *Nat. Commun.* 9:2794. 10.1038/s41467-018-05273-7 30022074 PMC6052026

[B12] Choi S. Jeong H. J. Kim H. Choi D. Cho S. C. Seong J. K. (2019). Skeletal muscle-specific Prmt1 deletion causes muscle atrophy via deregulation of the PRMT6-FOXO3 axis. *Autophagy* 15 1069–1081. 10.1080/15548627.2019.1569931 30653406 PMC6526864

[B13] DeJesus-Hernandez M. Mackenzie I. R. Boeve B. F. Boxer A. L. Baker M. Rutherford N. J. (2011). Expanded GGGGCC hexanucleotide repeat in noncoding region of C9ORF72 causes chromosome 9p-linked FTD and ALS. *Neuron* 72 245–256. 10.1016/j.neuron.2011.09.011 21944778 PMC3202986

[B14] Dhar S. Vemulapalli V. Patananan A. N. Huang G. L. Di Lorenzo A. Richard S. (2013). Loss of the major Type I arginine methyltransferase PRMT1 causes substrate scavenging by other PRMTs. *Sci. Rep.* 3:1311. 10.1038/srep01311 23419748 PMC3575585

[B15] Du Z. W. Chen H. Liu H. Lu J. Qian K. Huang C. L. (2015). Generation and expansion of highly pure motor neuron progenitors from human pluripotent stem cells. *Nat. Commun.* 6:6626. 10.1038/ncomms7626 25806427 PMC4375778

[B16] Farg M. A. Sundaramoorthy V. Sultana J. M. Yang S. Atkinson R. A. K. Levina V. (2014). C9ORF72, implicated in amytrophic lateral sclerosis and frontotemporal dementia, regulates endosomal trafficking. *Hum. Mol. Genet.* 23 3579–3595. 10.1093/hmg/ddu068 24549040 PMC4049310

[B17] Fomin V. Richard P. Hoque M. Li C. Gu Z. Fissore-O’Leary M. (2018). The C9ORF72 gene, implicated in amyotrophic lateral sclerosis and frontotemporal dementia, encodes a protein that functions in control of endothelin and glutamate signaling. *Mol. Cell. Biol.* 38 e00155–18. 10.1128/MCB.00155-18 30150298 PMC6206455

[B18] Freibaum B. D. Taylor J. P. (2017). The role of dipeptide repeats in C9ORF72-related ALS-FTD. *Front. Mol. Neurosci.* 10:35. 10.3389/fnmol.2017.00035 28243191 PMC5303742

[B19] Frick P. Sellier C. Mackenzie I. R. A. Cheng C. Y. Tahraoui-Bories J. Martinat C. (2018). Novel antibodies reveal presynaptic localization of C9orf72 protein and reduced protein levels in C9orf72 mutation carriers. *Acta Neuropathol. Commun.* 6:72. 10.1186/s40478-018-0579-0 30075745 PMC6091050

[B20] Garcia-Diaz A. Efe G. Kabra K. Patel A. Lowry E. R. Shneider N. A. (2020). Standardized reporter systems for purification and imaging of human pluripotent stem cell-derived motor neurons and other cholinergic cells. *Neuroscience* 450 48–56. 10.1016/j.neuroscience.2020.06.028 32615233 PMC7688562

[B21] Gendron T. F. Petrucelli L. (2018). Disease mechanisms of C9ORF72 repeat expansions. *Cold Spring Harb. Perspect. Med.* 8:a024224. 10.1101/cshperspect.a024224 28130314 PMC5880161

[B22] Gill A. L. Premasiri A. S. Vieira F. G. (2021). Hypothesis and theory: Roles of arginine methylation in C9orf72-mediated ALS and FTD. *Front. Cell. Neurosci.* 15:633668. 10.3389/fncel.2021.633668 33833668 PMC8021787

[B23] Gill A. L. Wang M. Z. Levine B. Premasiri A. Vieira F. G. (2019). Primary neurons and differentiated NSC-34 cells are more susceptible to arginine-rich ALS dipeptide repeat protein-associated toxicity than non-differentiated NSC-34 and CHO cells. *IJMS* 20:6238. 10.3390/ijms20246238 31835664 PMC6941034

[B24] Gittings L. M. Boeynaems S. Lightwood D. Clargo A. Topia S. Nakayama L. (2020). Symmetric dimethylation of poly-GR correlates with disease duration in C9orf72 FTLD and ALS and reduces poly-GR phase separation and toxicity. *Acta Neuropathol.* 139 407–410. 10.1007/s00401-019-02104-x 31832771 PMC6989575

[B25] Haghandish N. Baldwin R. M. Morettin A. Dawit H. T. Adhikary H. Masson J.-Y. (2019). PRMT7 methylates eukaryotic translation initiation factor 2α and regulates its role in stress granule formation. *Mol. Biol. Cell.* 30 778–793. 10.1091/mbc.E18-05-0330 30699057 PMC6589776

[B26] He L. Liang J. Chen C. Chen J. Shen Y. Sun S. (2022). C9orf72 functions in the nucleus to regulate DNA damage repair. *Cell Death Differ*. 30 716–730. 10.1038/s41418-022-01074-0 36220889 PMC9984389

[B27] Huang T. Yang Y. Song X. Wan X. Wu B. Sastry N. (2021). PRMT6 methylation of RCC1 regulates mitosis, tumorigenicity, and radiation response of glioblastoma stem cells. *Mol. Cell* 81 1276–1291.e9. 10.1016/j.molcel.2021.01.015 33539787 PMC7979509

[B28] Ikenaka K. Atsuta N. Maeda Y. Hotta Y. Nakamura R. Kawai K. (2019). Increase of arginine dimethylation correlates with the progression and prognosis of ALS. *Neurology* 92 e1868–e1877. 10.1212/WNL.0000000000007311 30867270

[B29] Ikenaka K. Maeda Y. Hotta Y. Nagano S. Yamada S. Ito D. (2022). Serum asymmetric dimethylarginine level correlates with the progression and prognosis of amyotrophic lateral sclerosis. *Eur. J. Neurol.* 29 1410–1416. 10.1111/ene.15254 35128793 PMC9305138

[B30] Kanekura K. Harada Y. Fujimoto M. Yagi T. Hayamizu Y. Nagaoka K. (2018). Characterization of membrane penetration and cytotoxicity of C9orf72-encoding arginine-rich dipeptides. *Sci. Rep.* 8:12740. 10.1038/s41598-018-31096-z 30143685 PMC6109075

[B31] Kramer N. J. Haney M. S. Morgens D. W. Jovičić A. Couthouis J. Li A. (2018). CRISPR–Cas9 screens in human cells and primary neurons identify modifiers of C9ORF72 dipeptide-repeat-protein toxicity. *Nat. Genet.* 50 603–612. 10.1038/s41588-018-0070-7 29507424 PMC5893388

[B32] Kwon I. Xiang S. Kato M. Wu L. Theodoropoulos P. Wang T. (2014). Poly-dipeptides encoded by the C9orf72 repeats bind nucleoli, impede RNA biogenesis, and kill cells. *Science* 345 1139–1145. 10.1126/science.1254917 25081482 PMC4459787

[B33] Lafarga V. Sirozh O. Díaz-López I. Galarreta A. Hisaoka M. Zarzuela E. (2021). Widespread displacement of DNA- and RNA-binding factors underlies toxicity of arginine-rich cell-penetrating peptides. *EMBO J.* 40:e103311. 10.15252/embj.2019103311 33978236 PMC8246256

[B34] Lagier-Tourenne C. Baughn M. Rigo F. Sun S. Liu P. Li H. R. (2013). Targeted degradation of sense and antisense C9orf72 RNA foci as therapy for ALS and frontotemporal degeneration. *Proc. Natl. Acad. Sci. U. S. A.* 110 E4530–E4539. 10.1073/pnas.1318835110 24170860 PMC3839752

[B35] Lawson B. R. Manenkova Y. Ahamed J. Chen X. Zou J. P. Baccala R. (2007). Inhibition of transmethylation down-regulates CD4 T cell activation and curtails development of autoimmunity in a model system. *J. Immunol.* 178 5366–5374. 10.4049/jimmunol.178.8.5366 17404322

[B36] Lee K. H. Zhang P. Kim H. J. Mitrea D. M. Sarkar M. Freibaum B. D. (2016). C9orf72 dipeptide repeats impair the assembly, dynamics, and function of membrane-less organelles. *Cell* 167 774–788.e17. 10.1016/j.cell.2016.10.002 27768896 PMC5079111

[B37] Leskelä S. Huber N. Rostalski H. Natunen T. Remes A. Takalo M. (2019). C9orf72 proteins regulate autophagy and undergo autophagosomal or proteasomal degradation in a cell type-dependent manner. *Cells* 8:1233. 10.3390/cells8101233 31658762 PMC6829620

[B38] Liu Y. Andreucci A. Iwamoto N. Yin Y. Yang H. Liu F. (2022). Preclinical evaluation of WVE-004, an investigational stereopure oligonucleotide for the treatment of C9orf72-associated ALS or FTD. *Mol. Ther. Nucleic Acids* 28 558–570. 10.1016/j.omtn.2022.04.007 35592494 PMC9092894

[B39] Liu Y. Dodart J. C. Tran H. Berkovitch S. Braun M. Byrne M. (2021). Variant-selective stereopure oligonucleotides protect against pathologies associated with C9orf72-repeat expansion in preclinical models. *Nat. Commun.* 12:847. 10.1038/s41467-021-21112-8 33558503 PMC7870851

[B40] Liu Y. Huang Z. Liu H. Ji Z. Arora A. Cai D. (2023). DNA-initiated epigenetic cascades driven by C9orf72 hexanucleotide repeat. *Neuron* 10.1016/j.neuron.2023.01.022 [Epub ahead of print]. 37080168 PMC10197862

[B41] Lorton B. M. Shechter D. (2019). Cellular consequences of arginine methylation. *Cell. Mol. Life Sci.* 76 2933–2956. 10.1007/s00018-019-03140-2 31101937 PMC6642692

[B42] Majounie E. Renton A. E. Mok K. Dopper E. G. Waite A. Rollinson S. (2012). Frequency of the C9orf72 hexanucleotide repeat expansion in patients with amyotrophic lateral sclerosis and frontotemporal dementia: A cross-sectional study. *Lancet Neurol.* 11 323–330. 10.1016/S1474-4422(12)70043-1 22406228 PMC3322422

[B43] Maron M. I. Lehman S. M. Gayatri S. DeAngelo J. D. Hegde S. Lorton B. M. (2021). Independent transcriptomic and proteomic regulation by type I and II protein arginine methyltransferases. *iScience* 24:102971. 10.1016/j.isci.2021.102971 34505004 PMC8417332

[B44] Masrori P. Van Damme P. (2020). Amyotrophic lateral sclerosis: A clinical review. *Eur. J. Neurol.* 27 1918–1929. 10.1111/ene.14393 32526057 PMC7540334

[B45] McGoldrick P. Lau A. You Z. Durcan T. M. Robertson J. (2023). Loss of C9orf72 perturbs the Ran-GTPase gradient and nucleocytoplasmic transport, generating compositionally diverse Importin β-1 granules. *Cell Rep.* 42:112134. 10.1016/j.celrep.2023.112134 36821445

[B46] Millar S. R. Huang J. Q. Schreiber K. J. Tsai Y. C. Won J. Zhang J. (2023). A new phase of networking: The molecular composition and regulatory dynamics of mammalian stress granules. *Chem. Rev.* 10.1021/acs.chemrev.2c00608 [Epub ahead of print]. 36662637 PMC10375481

[B47] Mori K. Weng S. M. Arzberger T. May S. Rentzsch K. Kremmer E. (2013). The C9orf72 GGGGCC repeat is translated into aggregating dipeptide-repeat proteins in FTLD/ALS. *Science* 339 1335–1338. 10.1126/science.1232927 23393093

[B48] O’Rourke J. G. Bogdanik L. Yáñez A. Lall D. Wolf A. J. Muhammad A. K. M. G. (2016). C9orf72 is required for proper macrophage and microglial function in mice. *Science* 351 1324–1329. 10.1126/science.aaf1064 26989253 PMC5120541

[B49] Pal A. Kretner B. Abo-Rady M. Glaβ H. Dash B. P. Naumann M. (2021). Concomitant gain and loss of function pathomechanisms in C9ORF72 amyotrophic lateral sclerosis. *Life Sci. Alliance* 4:e202000764. 10.26508/lsa.202000764 33619157 PMC7918691

[B50] Peng C. Wong C. C. (2017). The story of protein arginine methylation: Characterization, regulation, and function. *Expert Rev. Proteom.* 14 157–170. 10.1080/14789450.2017.1275573 28043171

[B51] Premasiri A. S. Gill A. L. Vieira F. G. (2020). Type I PRMT inhibition protects against C9ORF72 arginine-rich dipeptide repeat toxicity. *Front. Pharmacol.* 11:569661. 10.3389/fphar.2020.569661 33013410 PMC7508178

[B52] Renton A. E. Majounie E. Waite A. Simon-Sanchez J. Rollinson S. Gibbs J. R. (2011). A hexanucleotide repeat expansion in C9ORF72 is the cause of chromosome 9p21-Linked ALS-FTD. *Neuron* 72 257–268. 10.1016/j.neuron.2011.09.010 21944779 PMC3200438

[B53] Sellier C. Campanari M. L. Corbier C. Gaucherot A. Kolb-Cheynel I. Oulad-Abdelghani M. (2016). Loss of C9orf72 impairs autophagy and synergizes with polyQ Ataxin-2 to induce motor neuron dysfunction and cell death. *EMBO J.* 35 1276–1297. 10.15252/embj.201593350 27103069 PMC4910533

[B54] Shi Y. Lin S. Staats K. A. Li Y. Chang W. H. Hung S. T. (2018). Haploinsufficiency leads to neurodegeneration in C9ORF72 ALS/FTD human induced motor neurons. *Nat. Med.* 24 313–325. 10.1038/nm.4490 29400714 PMC6112156

[B55] Sivadasan R. Hornburg D. Drepper C. Frank N. Jablonka S. Hansel A. (2016). C9ORF72 interaction with cofilin modulates actin dynamics in motor neurons. *Nat. Neurosci.* 19 1610–1618. 10.1038/nn.4407 27723745

[B56] Smith W. A. Schurter B. T. Wong-Staal F. David M. (2004). Arginine methylation of RNA helicase A determines Its subcellular localization. *J. Biol. Chem.* 279 22795–22798. 10.1074/jbc.C300512200 15084609

[B57] Stouth D. W. vanLieshout T. L. Shen N. Y. Ljubicic V. (2017). Regulation of skeletal muscle plasticity by protein arginine methyltransferases and their potential roles in neuromuscular disorders. *Front. Physiol.* 8:870. 10.3389/fphys.2017.00870 29163212 PMC5674940

[B58] Suh E. Lee E. B. Neal D. Wood E. M. Toledo J. B. Rennert L. (2015). Semi-automated quantification of C9orf72 expansion size reveals inverse correlation between hexanucleotide repeat number and disease duration in frontotemporal degeneration. *Acta Neuropathol.* 130 363–372. 10.1007/s00401-015-1445-9 26022924 PMC4545720

[B59] Sullivan P. M. Zhou X. Robins A. M. Paushter D. H. Kim D. Smolka M. B. (2016). The ALS/FTLD associated protein C9orf72 associates with SMCR8 and WDR41 to regulate the autophagy-lysosome pathway. *Acta Neuropathol. Commun.* 4:51. 10.1186/s40478-016-0324-5 27193190 PMC4870812

[B60] Tao Z. Wang H. Xia Q. Li K. Li K. Jiang X. (2015). Nucleolar stress and impaired stress granule formation contribute to C9orf72 RAN translation-induced cytotoxicity. *Hum. Mol. Genet.* 24 2426–2441. 10.1093/hmg/ddv005 25575510

[B61] Tran H. Moazami M. P. Yang H. McKenna-Yasek D. Douthwright C. L. Pinto C. (2022). Suppression of mutant C9orf72 expression by a potent mixed backbone antisense oligonucleotide. *Nat. Med.* 28 117–124. 10.1038/s41591-021-01557-6 34949835 PMC8861976

[B62] Tsai W. C. Gayatri S. Reineke L. C. Sbardella G. Bedford M. T. Lloyd R. E. (2016). Arginine demethylation of G3BP1 promotes stress granule assembly. *J. Biol. Chem.* 291 22671–22685. 10.1074/jbc.M116.739573 27601476 PMC5077203

[B63] Tsang B. Arsenault J. Vernon R. M. Lin H. Sonenberg N. Wang L.-Y. (2019). Phosphoregulated FMRP phase separation models activity-dependent translation through bidirectional control of mRNA granule formation. *Proc. Natl. Acad. Sci. U. S. A.* 116 4218–4227. 10.1073/pnas.1814385116 30765518 PMC6410804

[B64] Urulangodi M. Mohanty A. (2020). DNA damage response and repair pathway modulation by non-histone protein methylation: Implications in neurodegeneration. *J. Cell Commun. Signal.* 14 31–45. 10.1007/s12079-019-00538-2 31749026 PMC7176765

[B65] van Blitterswijk M. Gendron T. F. Baker M. C. DeJesus-Hernandez M. Finch N. A. Brown P. H. (2015). Novel clinical associations with specific C9ORF72 transcripts in patients with repeat expansions in C9ORF72. *Acta Neuropathol.* 130 863–876. 10.1007/s00401-015-1480-6 26437865 PMC4655160

[B66] Van Mossevelde S. van der Zee J. Cruts M. Van Broeckhoven C. (2017). Relationship between C9orf72 repeat size and clinical phenotype. *Curr. Opin. Genet. Dev.* 44 117–124. 10.1016/j.gde.2017.02.008 28319737

[B67] Waite A. J. Bäumer D. East S. Neal J. Morris H. R. Ansorge O. (2014). Reduced C9orf72 protein levels in frontal cortex of amyotrophic lateral sclerosis and frontotemporal degeneration brain with the C9ORF72 hexanucleotide repeat expansion. *Neurobiol. Aging* 35 1779.e5–1779.e13. 10.1016/j.neurobiolaging.2014.01.016 24559645 PMC3988882

[B68] Webster C. P. Smith E. F. Bauer C. S. Moller A. Hautbergue G. M. Ferraiuolo L. (2016). The C9orf72 protein interacts with Rab1a and the ULK 1 complex to regulate initiation of autophagy. *EMBO J.* 35 1656–1676. 10.15252/embj.201694401 27334615 PMC4969571

[B69] Wen X. Tan W. Westergard T. Krishnamurthy K. Markandaiah S. S. Shi Y. (2014). Antisense proline-arginine ran dipeptides linked to C9ORF72-ALS/FTD form toxic nuclear aggregates that initiate in vitro and in vivo neuronal death. *Neuron* 84 1213–1225. 10.1016/j.neuron.2014.12.010 25521377 PMC4632245

[B70] Xiao S. MacNair L. McLean J. McGoldrick P. McKeever P. Soleimani S. (2016). C9orf72 isoforms in amyotrophic lateral sclerosis and frontotemporal lobar degeneration. *Brain Res.* 1647 43–49. 10.1016/j.brainres.2016.04.062 27134035

[B71] Xie W. Denman R. B. (2011). Protein methylation and stress granules: Posttranslational remodeler or innocent bystander? *Mol. Biol. Int.* 2011:137459. 10.4061/2011/137459 22091395 PMC3196864

[B72] Yang M. Liang C. Swaminathan K. Herrlinger S. Lai F. Shiekhattar R. (2016). A C9ORF72/SMCR8-containing complex regulates ULK1 and plays a dual role in autophagy. *Sci. Adv.* 2:e1601167. 10.1126/sciadv.1601167 27617292 PMC5010369

[B73] Yang Q. Jiao B. Shen L. (2020). The development of C9orf72-related amyotrophic lateral sclerosis and frontotemporal dementia disorders. *Front. Genet.* 11:562758. 10.3389/fgene.2020.562758 32983232 PMC7492664

[B74] Zampatti S. Peconi C. Campopiano R. Gambardella S. Caltagirone C. Giardina E. (2022). C9orf72-related neurodegenerative diseases: From clinical diagnosis to therapeutic strategies. *Front. Aging Neurosci.* 14:907122. 10.3389/fnagi.2022.907122 35754952 PMC9226392

[B75] Zheng W. Wang K. Wu Y. Yan G. Zhang C. Li Z. (2022). C9orf72 regulates the unfolded protein response and stress granule formation by interacting with eIF2α. *Theranostics* 12 7289–7306. 10.7150/thno.76138 36438488 PMC9691347

[B76] Zhu Q. Jiang J. Gendron T. F. McAlonis-Downes M. Jiang L. Taylor A. (2020). Reduced C9ORF72 function exacerbates gain of toxicity from ALS/FTD-causing repeat expansion in C9orf72. *Nat. Neurosci.* 23 615–624. 10.1038/s41593-020-0619-5 32284607 PMC7384305

[B77] Zu T. Guo S. Bardhi O. Ryskamp D. A. Li J. Khoramian Tusi S. (2020). Metformin inhibits RAN translation through PKR pathway and mitigates disease in C9orf72 ALS/FTD mice. *Proc. Natl. Acad. Sci. U. S. A.* 117 18591–18599. 10.1073/pnas.2005748117 32690681 PMC7414156

