# Supplementary material for: Reduced C9orf72 expression exacerbates polyGR toxicity in patient iPSC-derived motor neurons and a Type I protein arginine methyltransferase inhibitor reduces that toxicity
Source: Front Cell Neurosci. 2023 Apr 17;17:1134090. doi: 10.3389/fncel.2023.1134090 (PMC10149854; doi:10.3389/fncel.2023.1134090)
Supplement: Supplementary file 1 [file Data_Sheet_1.pdf]

frontiers

Supplementary Material

Reduced C9orf72 Expression Exacerbates polyGR Toxicity in Patient Induced Pluripotent Stem Cell-Derived Motor Neurons and a Type I Protein Arginine Methyltransferase Inhibitor Reduces that Toxicity

Therese L. Dane[1], Anna L. Gill[1], Fernando G. Vieira[1*], Kyle R. Denton[1]

[1]ALS Therapy Development Institute, Watertown, MA, USA

*** Correspondence:** Fernando G. Vieira: fvieira@als.net

1 Supplementary Figures

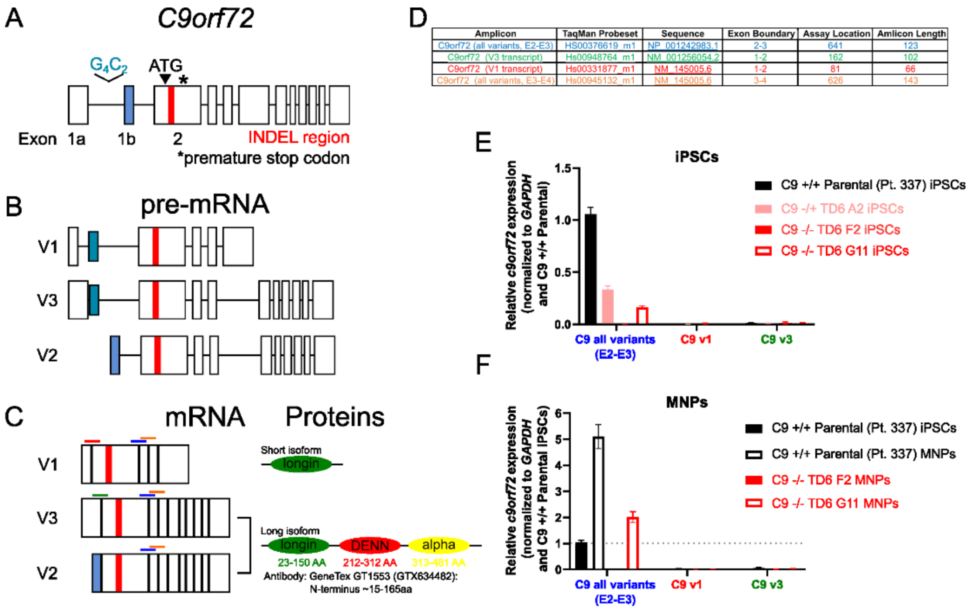

Supplementary Figure 1: Expression of *C9orf72* Splice Variants

(**A-C**) Schematic representation of the *C9orf72* gene, pre-mRNA, mRNA, and C9orf72 protein. CRISPR-Cas9 targeted region of exon 2 highlighted in red introduces a downstream premature stop codon. Antibody used in Fig. 1 binds the N-terminus of C9orf72. Modified from Liu et al. 2021 and Xiao et al. 2016. (**D**) Table of TaqMan probe sets used in E-F. (**E**) Expression of total *C9orf72*, transcript v1, and transcript v3 in iPSCs normalized to C9+/+ parental (Pt. 337). (**F**) Expression of *C9orf72* in motor neuron progenitors normalized to C9+/+ parental (Pt. 337) iPSCs.

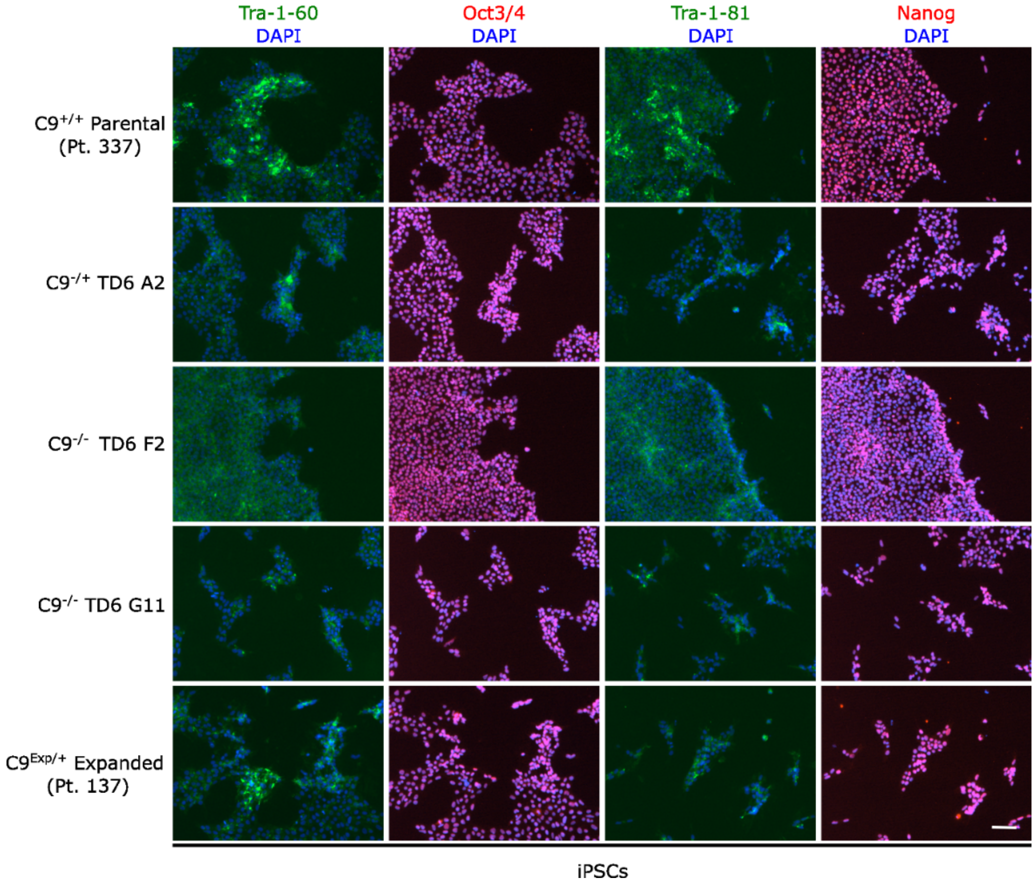

Supplementary Figure 2: *C9orf72* Knockout iPSCs Maintain Pluripotency Marker Expression

Representative images of immunocytochemistry for pluripotency markers Tra-1-60, Oct3/4, Tra-1-81, and Nanog.

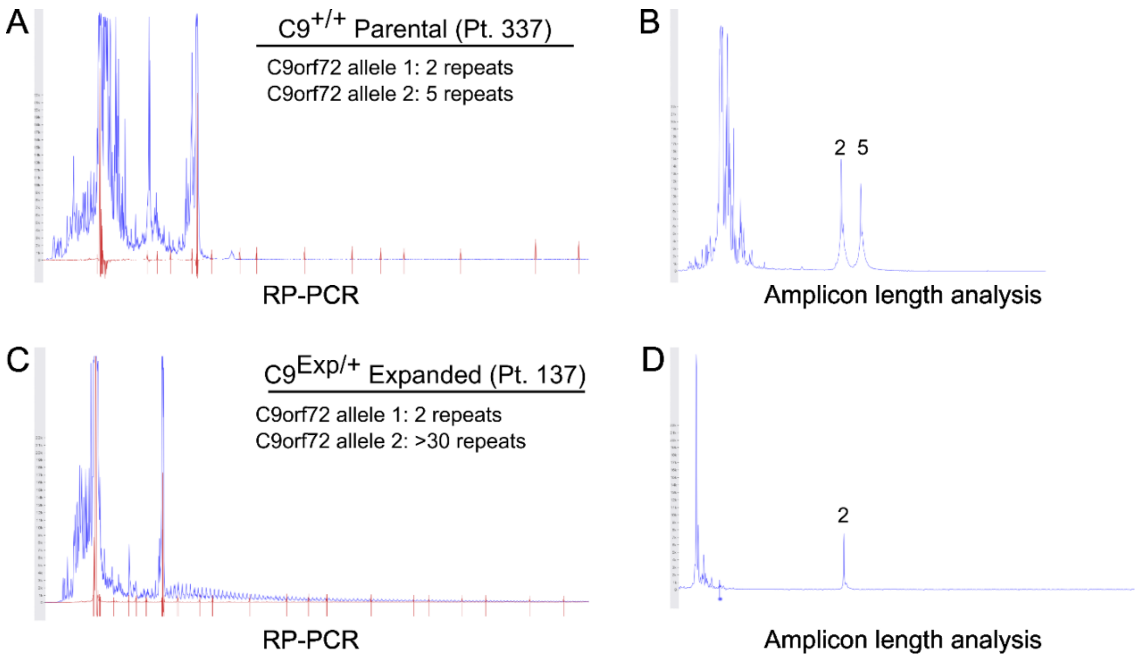

Supplementary Figure 3: *C9orf72*-HRE Genotyping by Repeat-Primed PCR

(**A**) Repeat-primed PCR electropherograms from patient 337 fibroblast genomic DNA. (**B**) Amplicon length analysis electropherograms from patient 337 fibroblast genomic DNA. (**C**) Repeat-primed PCR electropherograms from patient 137 fibroblast genomic DNA. Note the sawtooth pattern characteristic of long C9-HRE expansions. (**D**) Amplicon length analysis electropherograms from patient 137 fibroblast genomic DNA.

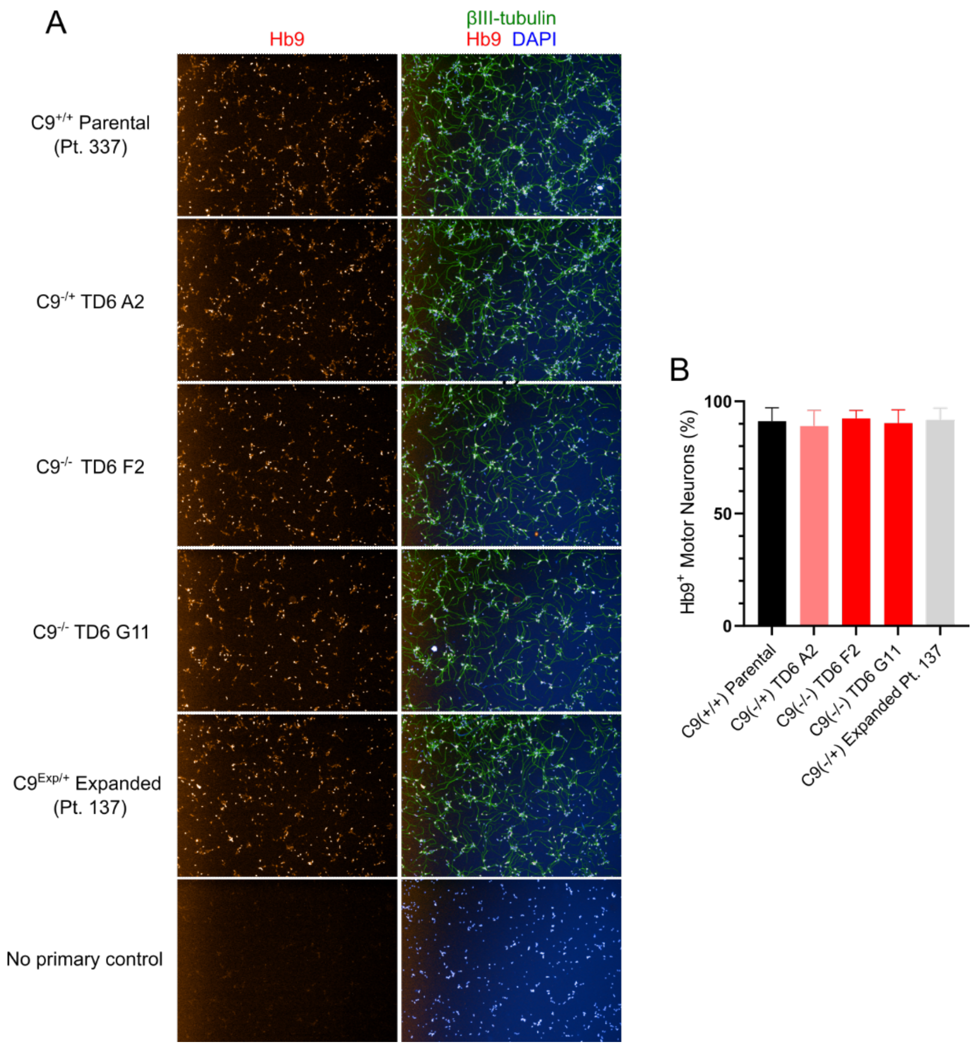

Supplementary Figure 4: Spinal Motor Neuron Differentiation Efficiency

(A) Representative images of iPSC-MNs stained on day 25 of differentiation for the pan-neuronal marker βIII-tubulin (Covance MRB-435P, 1:500) and the spinal motor neuron specific transcription factor Hb9 (*MNX1*, DSHB 815.c10c, 1;100). **(B)** Quantification of the percentage of Hb9^{+} cells. No significant difference was observed between cell lines.

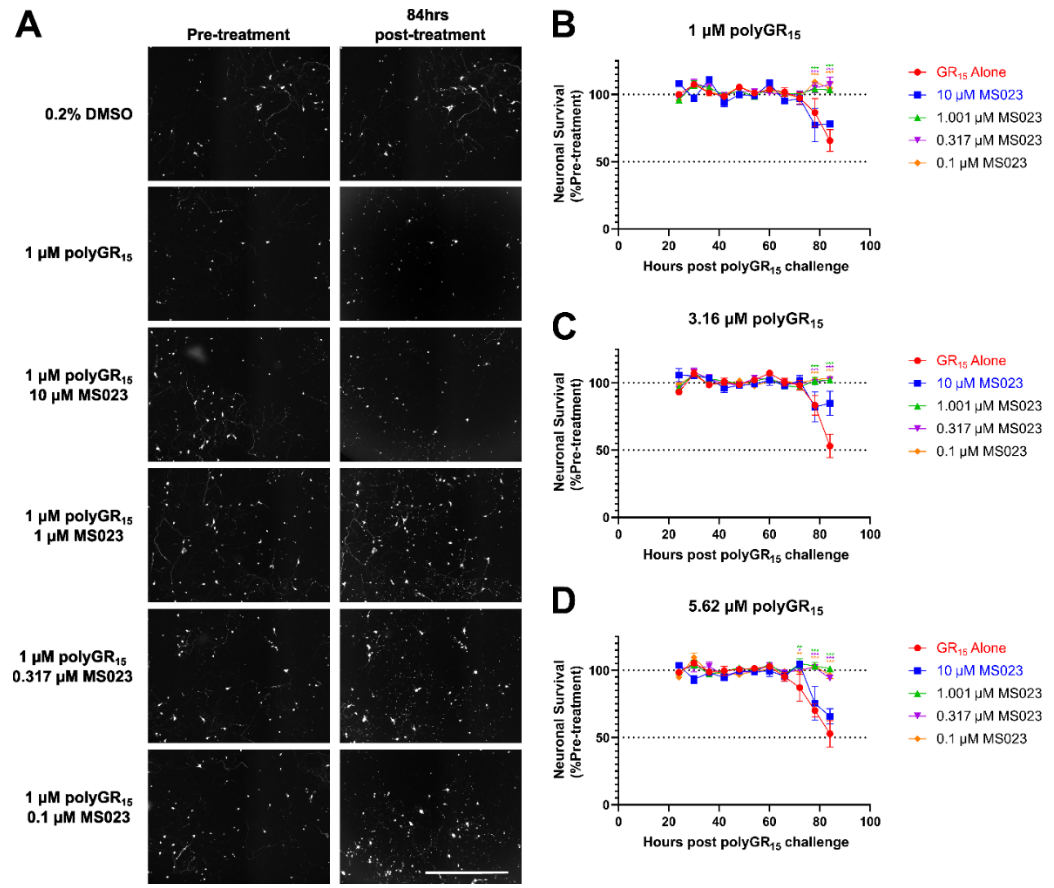

Supplementary Figure 5: Type I PRMT Inhibition Preserves Neuronal Survival

(**A**) Representative images of day 12 VACHT-tdTomato neurons before and after polyGR$_{15}$ challenge with or without MS023 co-treatment. Scale bar = 2000 µm. (**B-D**) Quantification of the number of tdTomato^{+} MNs measured every 6 hours beginning 24 hours post treatment. For each well, the neuron count was normalized to the same wells previous time point. * denotes $p < 0.05$, ** denotes $p < 0.01$, *** denotes $p < 0.001$ (comparing MS023 co-treated groups to polyGR$_{15}$ alone by two-way ANOVA analysis of variance followed by Dunnett's test). Data are presented as mean values ± standard deviations (error bars).

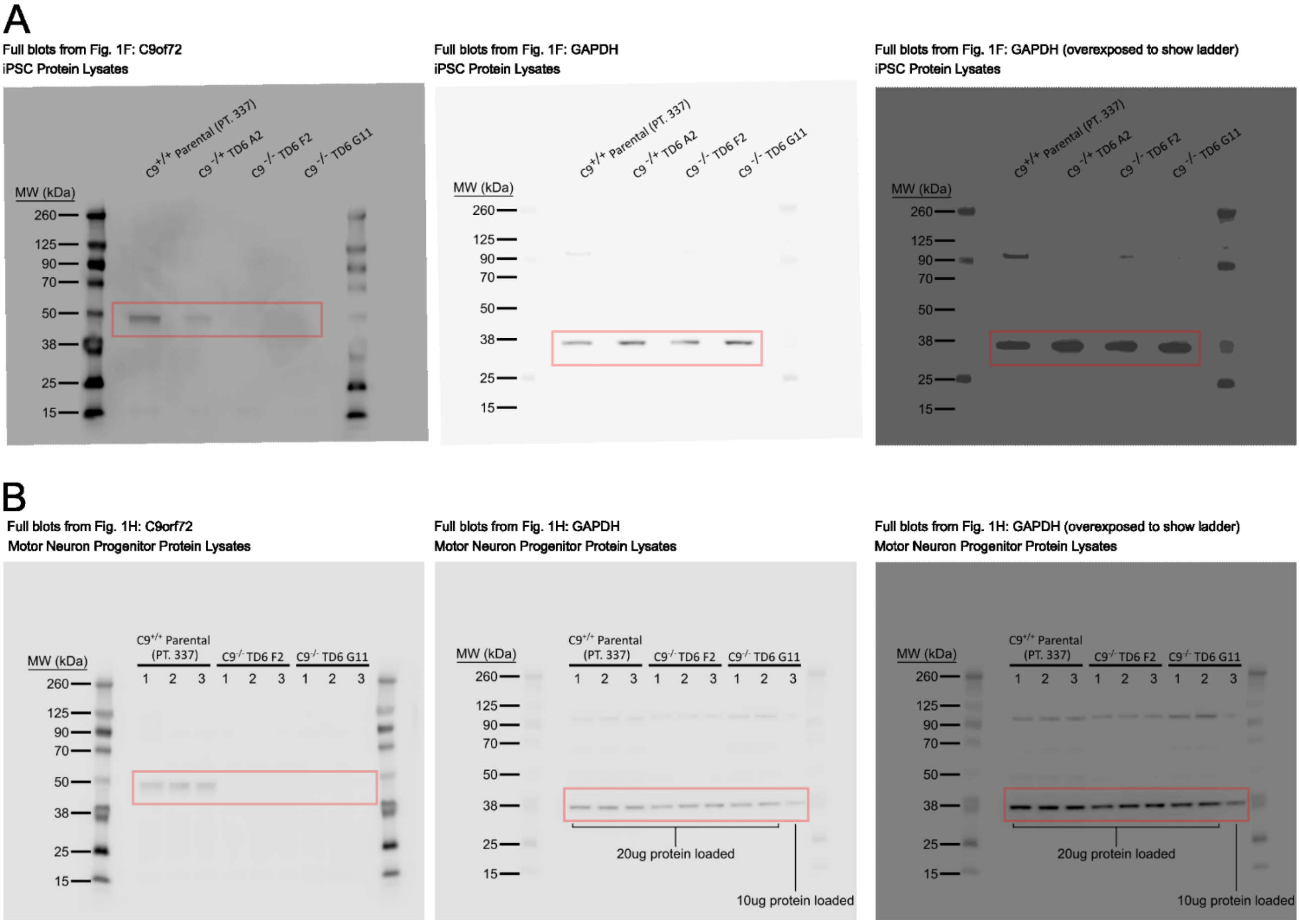

Supplementary Figure 6: Full Uncropped Western Blot Images from Figure 1

(A) Full blots for C9orf72 and GAPDH from iPSC lysates shown in Fig. 1F. **(B)** Full blots for C9orf72 and GAPDH from iPSC lysates shown in Fig. 1H. Only 10ug of total protein was loaded for lane 3 of C9$^{-/-}$ TD6 G11 due to insufficient sample volume.
